# Concurrent activation of growth factor and nutrient arms of mTORC1 induces oxidative liver injury

**DOI:** 10.1038/s41421-019-0131-9

**Published:** 2019-11-19

**Authors:** Chun-Seok Cho, Allison H. Kowalsky, Sim Namkoong, Sung-Rye Park, Shuangcheng Wu, Boyoung Kim, Amanda James, Bondong Gu, Ian A. Semple, Mohamed A. Tohamy, Sumeet Solanki, Uhn-Soo Cho, Joel K. Greenson, Yatrik M. Shah, Myungjin Kim, Jun Hee Lee

**Affiliations:** 1 Department of Molecular and Integrative Physiology, University of Michigan, Ann Arbor, MI 48109 USA; 2 Department of Biological Chemistry, University of Michigan, Ann Arbor, MI 48109 USA; 3 Department of Pathology, University of Michigan, Ann Arbor, MI 48109 USA; 4 Present Address: Department of Biochemistry, College of Natural Sciences, Kangwon National University, Chuncheon, Gangwon 24341 Republic of Korea; 5 Present Address: Department of Biochemistry, Faculty of Pharmacy, Beni-Suef University, Beni-Suef, Egypt

**Keywords:** Mechanisms of disease, TOR signalling, Transcriptomics

## Abstract

mTORC1 is a protein kinase important for metabolism and is regulated by growth factor and nutrient signaling pathways, mediated by the Rheb and Rag GTPases, respectively. Here we provide the first animal model in which both pathways were upregulated through concurrent mutations in their GTPase-activating proteins, *Tsc1* and *Depdc5*. Unlike former models that induced limited mTORC1 upregulation, hepatic deletion of both *Tsc1* and *Depdc5* (DKO) produced strong, synergistic activation of the mTORC1 pathway and provoked pronounced and widespread hepatocyte damage, leading to externally visible liver failure phenotypes, such as jaundice and systemic growth defects. The transcriptome profile of DKO was different from single knockout mutants but similar to those of diseased human livers with severe hepatitis and mouse livers challenged with oxidative stress-inducing chemicals. In addition, DKO liver cells exhibited prominent molecular pathologies associated with excessive endoplasmic reticulum (ER) stress, oxidative stress, DNA damage and inflammation. Although DKO liver pathologies were ameliorated by mTORC1 inhibition, ER stress suppression unexpectedly aggravated them, suggesting that ER stress signaling is not the major conduit of how hyperactive mTORC1 produces liver damage. Interestingly, superoxide scavengers N-acetylcysteine (NAC) and Tempol, chemicals that reduce oxidative stress, were able to recover liver phenotypes, indicating that mTORC1 hyperactivation induced liver damage mainly through oxidative stress pathways. Our study provides a new model of unregulated mTORC1 activation through concomitant upregulation of growth factor and nutrient signaling axes and shows that mTORC1 hyperactivation alone can provoke oxidative tissue injury.

## Introduction

Mammalian target of rapamycin complex 1 (mTORC1) is a protein kinase complex that promotes cellular anabolism in response to insulin/growth factor stimuli and nutrient abundance^1–4^. Regulation of mTORC1 is believed to be mediated by two small G proteins, Rheb and Rag^4,5^. The tuberous sclerosis complex (TSC) and the GAP activities Towards Rags 1 complex (GATOR1) are GTPase-activating proteins (GAPs) that regulate Rheb and Rag, respectively^4,5^. TSC, consisting of the TSC1 and TSC2 proteins, mediates growth factor and energy signals to mTORC1^6,7^, while GATOR1, consisting of DEPDC5, NPRL2 and NPRL3 proteins are essential for amino acid sensing^8,9^ and stress response^10^ of the mTORC1 pathway.

DEPDC5 is a component of GATOR1 that is critical for binding and inhibiting Rag^8,9^. DEPDC5 is also implicated in various human pathologies including brain and liver diseases^11–15^. Genetic variations in the *DEPDC5* locus were associated with hepatitis C virus (HCV)-induced hepatocellular carcinoma in a Japanese population^13^, HCV-induced fibrosis progression in a European population^14^, and hepatitis B virus (HBV)-related hepatocarcinogenesis in a Chinese population^15^. However, whether DEPDC5 regulates liver homeostasis and how it affects liver disease progression has not been investigated in an intact animal model.

mTORC1, the DEPDC5 and TSC1 target, is an important metabolic regulator in the liver^2,3^. mTORC1 activation is important for upregulating protein translation by phosphorylating two substrates: p70 ribosomal protein S6 kinase (S6K) and translation initiation factor 4E-binding protein 1 (4E-BP1)^1^. mTORC1 also upregulates lipid and nucleic acid synthesis while downregulating autophagic catabolism through inhibition of unc-51-like autophagy activating kinase (ULK1)^1–4^. Therefore, mTORC1 regulation is thought to be critical for maintaining metabolic homeostasis in the liver^2,3^. Indeed, disrupting mTORC1 through liver-specific deletion of Raptor, an essential subunit, induced spontaneous liver damage associated with inflammation and fibrosis^16^. This accelerated liver carcinogenesis upon administration of diethylnitrosamine (DEN), a chemical hepatocarcinogen^16^. Activating mTORC1 through hepatocyte-specific deletion of *Tsc1* (*Tsc1*^*Δhep*^) also produced liver inflammation and carcinogenesis in aged mice, but these pathologies were not obvious in young mice^17,18^.

Given the importance of DEPDC5 in nutrient and stress-dependent mTORC1 regulation^8–10^, DEPDC5 could be an important regulator of mTORC1 in hepatocytes. To understand the genetic role of DEPDC5 in the liver, we generated *Depdc5*^*Δhep*^ mice, which have hepatocyte-specific deletion of the *Depdc5* gene. Similar to *Tsc1*^*Δhep*^ mice, *Depdc5*^*Δhep*^ mice showed slight elevation in mTORC1 activity and exhibited mild inflammation and fibrosis in advanced age. However, when *Depdc5*^*Δhep*^ mice were crossed to *Tsc1*^*Δhep*^ mice, a much more striking phenotype was observed. Although individual deletions of *Depdc5* or *Tsc1* in the liver only slightly upregulated mTORC1 with no gross phenotypes, hepatocyte-specific *Depdc5* and *Tsc1* double knockout (DKO) mice had robust mTORC1 activation that induced prominent hepatocyte damage. Consequently, serious liver failure associated with jaundice, hepatomegaly, fur discoloration and growth suppression were observed by 8 weeks of age. Transcriptomic analyses with RNA-seq and subsequent protein analyses indicated that DKO livers suffer excessive ER stress and oxidative stress leading to metabolic dysregulation, DNA damage and inflammation. Among these outputs, oxidative damage was the most critical in producing DKO pathologies, while ER stress signaling protected hepatocytes by suppressing mTORC1 in a negative feedback mechanism.

## Results

### Hepatic loss of *Depdc5* induces hepatocellular hypertrophy in zone 3

**Fig. 1:**
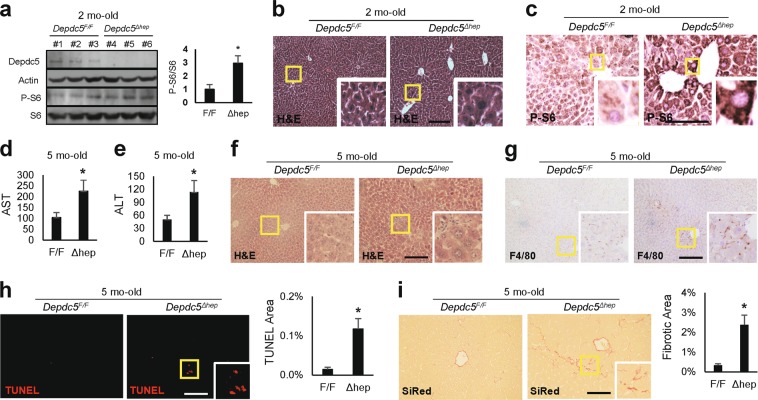
Liver-specific *Depdc5* deletion induces slight upregulation of mTORC1 and inflammation. Two-month-old **a**–**c** or five-month-old **d**–**i** littermates of *Depdc5*^*Δhep*^ and *Depdc5*^*F/F*^ male mice were subjected to the following analyses. **a** Liver lysates were subjected to immunoblotting with indicated antibodies (left). Band intensities were quantified (*n* = 3; right). **b**, **c** Liver sections were subjected to H&E staining **b** and anti-phospho-S6 immunostaining **c**. Boxed areas are magnified in the insets. **d**, **e** Serum AST **d** and ALT **e** assays (*n* ≥ 6). **f**–**i** Liver sections were subjected to H&E **f**, F4/80 **g**, TUNEL **h** and Sirius Red (SiRed, **i**) staining. TUNEL and SiRed-positive areas were quantified (*n* = 3). Data are shown as mean ± SEM. *P < 0.05 (Student’s *t*-test). Scale bars, 200 µm

Immunoblot analyses of two-month-old mouse liver indicated that *Alb-Cre*/*Depdc5*^*F/F*^ (*Depdc5*^*Δhep*^) mice lost hepatic Depdc5 expression and slightly upregulated the level of phosphorylated S6 (p-S6), a downstream marker of mTORC1 (Fig. 1a). Hematoxylin and eosin (H&E) staining of liver sections revealed that two-month-old *Depdc5*^*Δhep*^ mice had specific enlargement of pericentral zone 3 hepatocytes (Fig. 1b and Supplementary Fig. S1a), associated with locally elevated levels of p-S6 immunostaining (Fig. 1c and Supplementary Fig. S1a).

Consistent with impaired zone 3 homeostasis, *Depdc5*^*Δhep*^ mice were more extensively damaged from a high dose of acetaminophen (APAP), which provokes hepatocellular death most prominently in zone 3, compared to littermate controls (Supplementary Fig. S1b). APAP-induced hepatic mTORC1 activation^19–21^ was also stronger in *Depdc5*^*Δhep*^ mice (Supplementary Fig. S1c). Therefore, Depdc5 appears to be critical for homeostatic regulation of zone 3 hepatocytes, suppressing hepatic mTORC1 activation and hepatocellular hypertrophy, and protecting from APAP injury.

### *Depdc5*^*Δhep*^ mice exhibit mild zone 3 inflammation as they age

Five-month-old *Depdc5*^*Δhep*^ mice demonstrated a slight but significant elevation in serum markers of liver damage: AST (Fig. 1d) and ALT (Fig. 1e). Although these values are still within normal clinical ranges, it is possible that there are subclinical levels of mild liver pathologies. Histological analyses indeed revealed occasional liver inflammation (Fig. 1f, g), hepatocyte death (Fig. 1h) and fibrosis (Fig. 1i) in five-month-old *Depdc5*^*Δhep*^ mice. Immunoblot analyses also confirmed mTORC1 signaling upregulation (Supplementary Fig. S1d) and increased fibrogenic marker expression in five-month-old *Depdc5*^*Δhep*^ mice (Supplementary Fig. S1e). Therefore, similar to previously described *Tsc1*^*Δhep*^ mice^18^, *Depdc5*^*Δhep*^ mice also exhibited age-dependent development of spontaneous liver pathologies.

Despite inflammatory phenotypes, *Tsc1*^*Δhep*^ mice downregulated liver fat levels by blocking insulin-dependent lipogenic pathways^22^. Likewise, *Depdc5*^*Δhep*^ mice also exhibited reduced hepatic fat levels in both low fat diet (LFD, Supplementary Fig. S1f) and high fat diet (HFD, Supplementary Fig. S1g) conditions, without altering body weight gain (Supplementary Fig. S1h). Therefore, the phenotypes exhibited by liver-specific *Depdc5* knockouts were generally similar to *Tsc1* knockouts.

### Double deletion of *Tsc1* and *Depdc5* in liver suppresses systemic growth

**Fig. 2:**
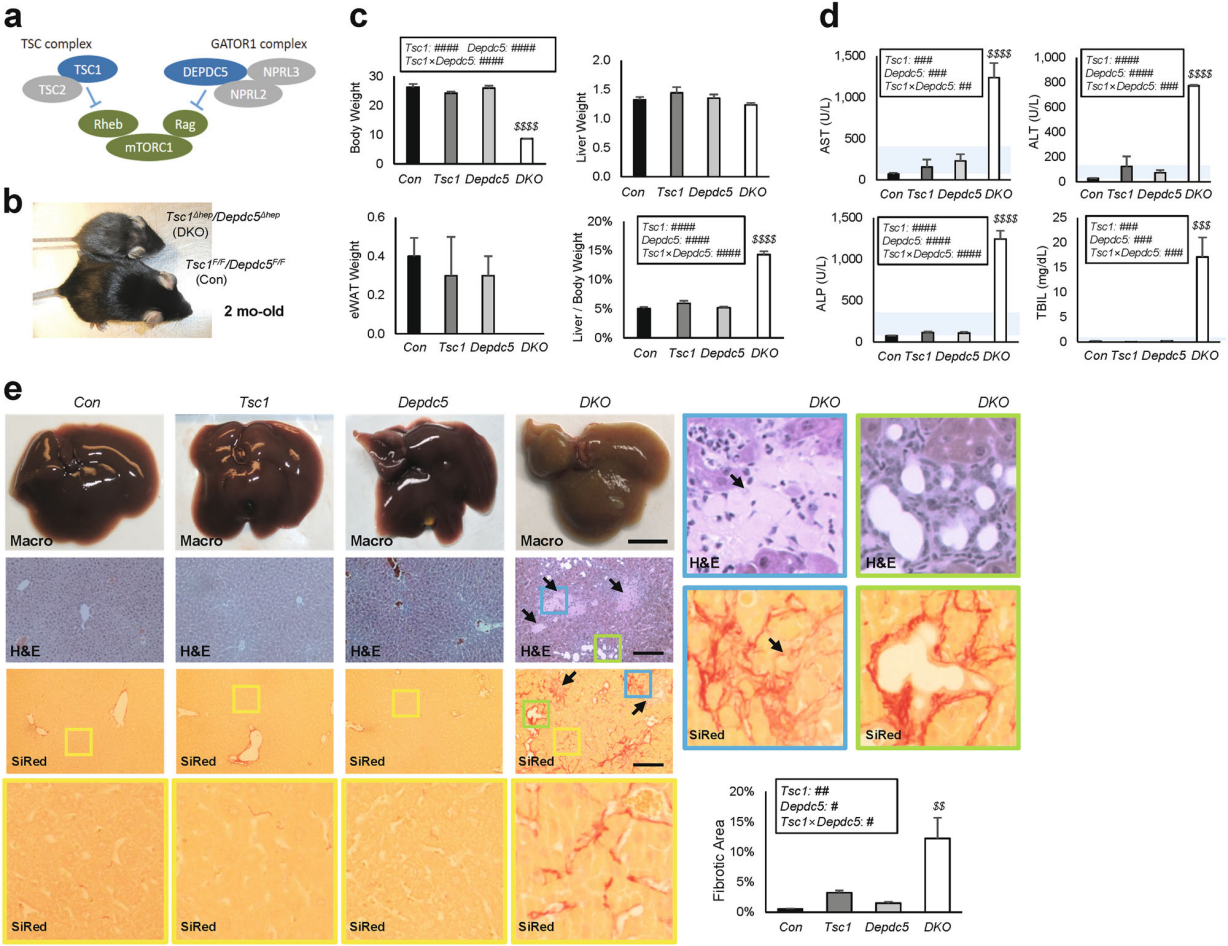
*Depdc5* and *Tsc1* mutations synergistically provoke liver injury and damage. Control (*Con*), *Tsc1*^*Δhep*^ (*Tsc1*), *Depdc5*^*Δhep*^ (*Depdc5*) and *Tsc1*^*Δhep*^/*Depdc5*^*Δhep*^ (*DKO*) male mice were generated as littermates and analyzed at two-months-old (*n* ≥ 3). **a** Schematic of how the Rheb and Rag pathways regulate mTORC1. **b** Gross appearance of *Con* and *DKO* littermates. **c** Body weight, liver weight, epididymal white adipose tissue (eWAT) weight and liver/body weight ratios. **d** Serum liver panel assays. Blue shaded regions indicate clinically normal ranges. **e** Macroscopic view of liver (Macro), H&E staining and Sirius Red (SiRed) staining of liver sections. Arrows indicate necrotic lesions. Yellow boxed areas are magnified in the corresponding bottom row. Blue and green boxed areas are magnified in right panels. Sirius Red-positive fibrotic areas were quantified. Data are shown as mean ± SEM. Effects of *Tsc1* and *Depdc5* mutations and their interaction (*Tsc1* × *Depdc5*) were assessed through two-way ANOVA (^#^*P* < 0.05; ^##^*P* < 0.01; ^###^*P* < 0.001; ^####^*P* < 0.0001), and statistical significance between *Con* and indicated groups were assessed through Tukey’s multiple comparison test (^*$$*^*P* < 0.01; ^*$$$*^*P* < 0.001; ^*$$$$*^*P* < 0.0001). Scale bars, 200 µm (histology) and 1 cm (whole liver)

Rag and Rheb are the two most important small GTPases directly regulating mTORC1^4,5^. Since Depdc5 and Tsc1 are critical for inhibiting Rag and Rheb, respectively, we hypothesized that mutations in these two genes may genetically interact (Fig. 2a). Even though these pathways were extensively studied in cultured cells, the genetic interaction between the Rag and Rheb pathways has not been examined in intact animals yet.

To test the genetic interaction, we crossed *Depdc5*^*Δhep*^ mice with *Tsc1*^*Δhep*^ mice. Although the *Depdc5*^*Δhep*^/*Tsc1*^*Δhep*^ double knockout (DKO) mice were born at the expected Mendelian ratios, their growth was severely suppressed, and their fur was gray and patchy by two months old (Fig. 2b, c and Supplementary Fig. S2a). These phenotypes were not observed in littermates of any other genotype, including *Depdc5*^*Δhep*^ and *Tsc1*^*Δhep*^ single knockouts. Although body and adipose tissue weights were drastically reduced in DKO mice, the liver weights were similar to controls and single knockout mice, resulting in a dramatic increase of liver/body weight ratio (Fig. 2c and Supplementary Fig. S2a).

The body weight difference between control and DKO mice was not observed in 6 day-old mice (Supplementary Fig. S2b), indicating that the DKO mice were not born with lower body weight and likely lose weight due to disease progression.

### DKO mice experience severe liver injury and failure

Sera from the DKO mice were yellow, indicating bilirubin accumulation. All serum markers for liver damage and dysfunction were elevated prominently above normal clinical ranges (Fig. 2d). Consistent with this, H&E staining revealed numerous necrotic lesions (arrows in Fig. 2e and Supplementary Fig. S2c) in DKO liver. The livers of DKO mice were extremely stiff, and Sirius Red staining revealed extensive pericellular fibrosis throughout the liver (Fig. 2e, bottom). Fibrotic lesions were more intense around necrotic regions and often associated with proliferating bile ducts (Fig. 2e, magnified images in blue and green boxes). All phenotypes were fully penetrant and prominently observed in both males (Fig. 2) and females (Supplementary Fig. S2a, c).

**Fig. 3:**
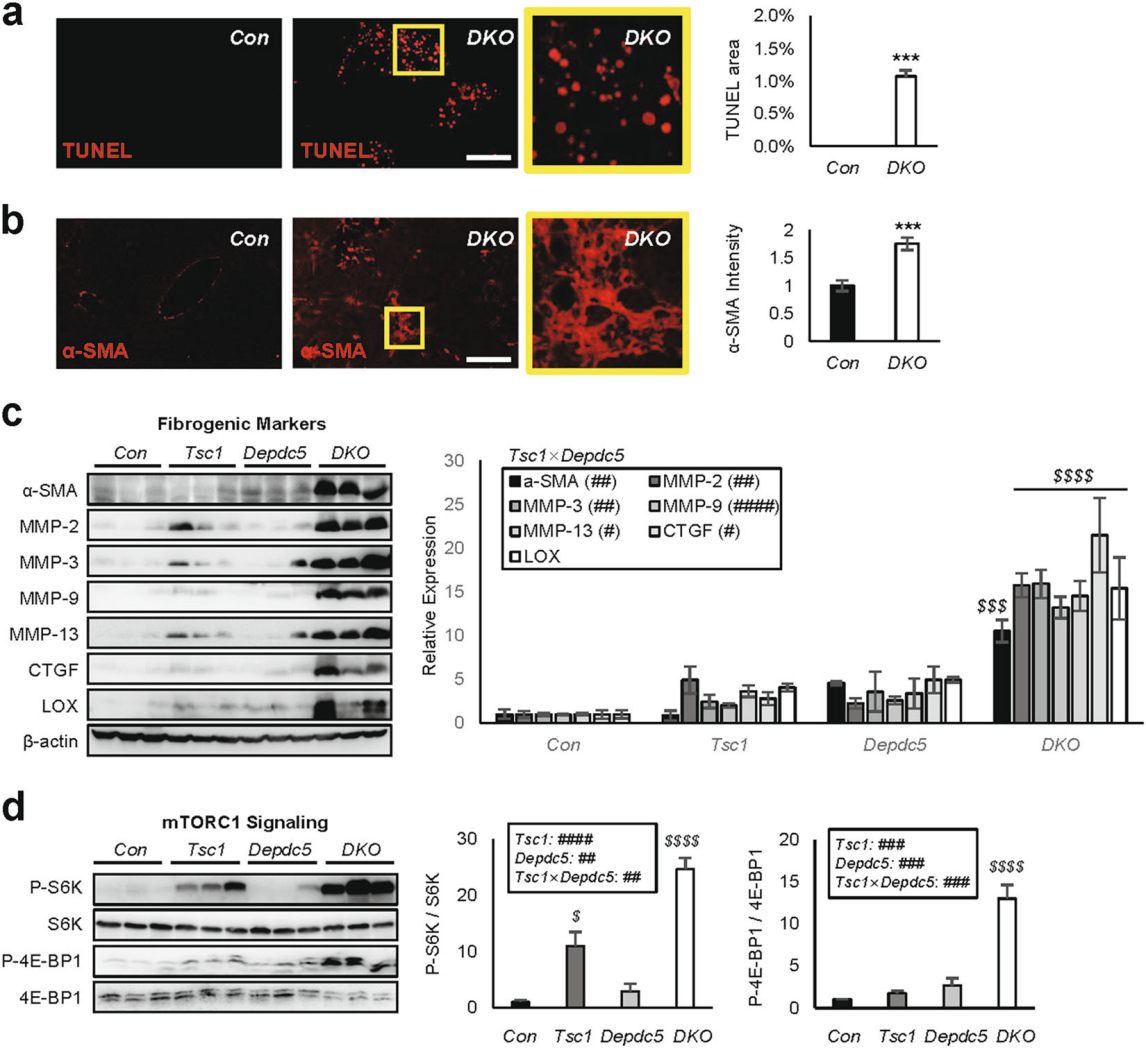
*Depdc5*/*Tsc1* double knockout (DKO) livers upregulate fibrosis and mTORC1 signaling. Mouse cohort described in Fig. 2 was subjected to histology **a**, **b** and immunoblotting **c**, **d** as outlined below. **a** TUNEL staining of liver sections. Boxed area is magnified in right panel. TUNEL-positive areas were quantified. **b** Liver sections were subjected to α-SMA staining. Boxed area was magnified in right panel. α-SMA staining intensities were quantified. **c** From the liver lysates, fibrogenic marker expression was analyzed through immunoblotting. Band intensities were quantified (*n* = 3). **d** From the liver lysates, phosphorylation of mTORC1 substrates were analyzed through immunoblotting. Band intensities were quantified (*n* = 3, mean ± SEM). Data are shown as mean ± SEM. ***P < 0.001 (Student’s *t*-test). Effects of *Tsc1* and *Depdc5* mutations and their interaction (*Tsc1* × *Depdc5*) were assessed through two-way ANOVA (^#^*P* < 0.05; ^##^*P* < 0.01; ^###^*P* < 0.001; ^####^*P* < 0.0001), and statistical significance between *Con* and indicated groups were assessed through Tukey’s multiple comparison test (^*$*^*P* < 0.05; ^*$$$*^*P* < 0.001; ^*$$$$*^*P* < 0.0001). Scale bars, 200 µm

Further characterization of liver tissues with TUNEL staining revealed increased apoptotic cells in DKO liver (Fig. 3a). In addition, both histology and immunoblot analyses confirmed that DKO livers had an increased expression of fibrogenic markers, significantly more than single knockouts (Fig. 3b, c). Then, we analyzed mTORC1 signaling by monitoring phosphorylation of its substrates, S6K and 4E-BP1. Although phosphorylation of these targets were upregulated in *Depdc5*^*Δhep*^ and *Tsc1*^*Δhep*^, DKO mouse liver exhibited synergistic activation, at levels far beyond the level achieved by single knockout littermates (Fig. 3d). This was not a simple, additive effect as the level of synergism was robust and statistically supported through two-way ANOVA (Fig. 3d). Therefore, concomitant activation of the Rheb and Rag pathways produced a strong genetic interaction and synergistically increased fibrosis and upregulated mTORC1 (Fig. 3).

### mTORC1 inhibition rescues DKO liver pathologies

**Fig. 4:**
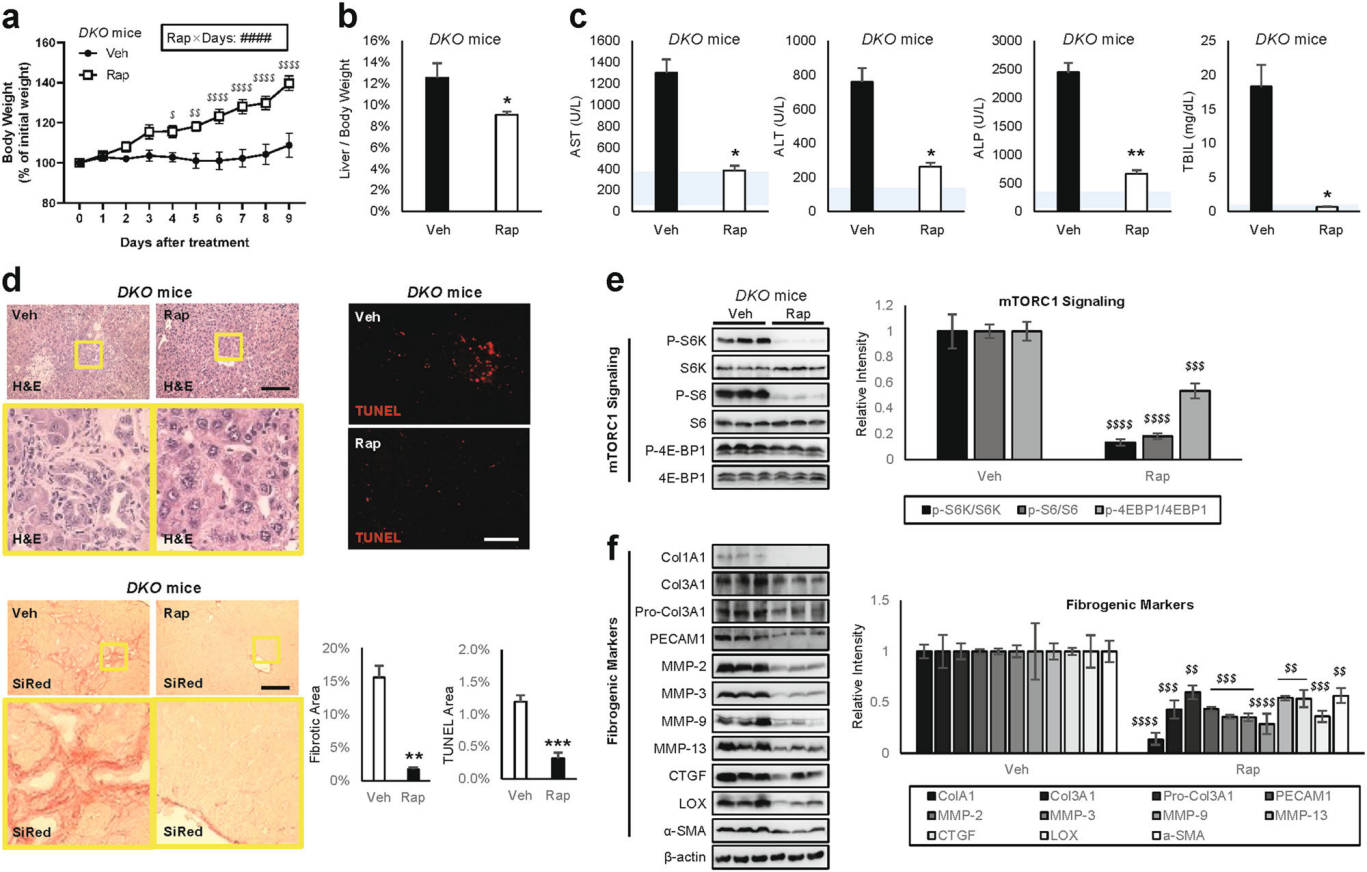
mTORC1 inhibition rescues DKO liver pathologies. Littermate cohorts of six-week-old DKO mice were injected daily with vehicle (Veh) or 10 mg/Kg rapamycin (Rap) for 10 days (*n* ≥ 4). For drug treatment experiments, mice were gender-matched with both males and females. **a** Body weight was monitored throughout the course of the experiment. **b** Liver/body weight ratio was measured at the experimental endpoint. **c** Serum markers for liver damage were analyzed. Blue shaded regions indicate clinically normal ranges. **d** Liver sections were analyzed by H&E, SiRed, and TUNEL staining. Boxed areas are magnified in lower panels. Fibrotic area and TUNEL area were quantified (*n* = 3). **e**, **f** Liver lysates were subjected to immunoblotting (left panels) and quantification (right panels) to examine mTORC1 signaling **e** and fibrogenic markers **f**. Data are shown as mean ± SEM. *P < 0.05; **P < 0.01; ***P < 0.001 (Student’s t-test). Interaction between rapamycin and treatment days (Rap × Days) were assessed through RM two-way ANOVA (^####^*P* < 0.0001), and differences in individual data points were assessed through Sidak’s multiple comparison test (^*$*^*P* < 0.05; ^*$$*^*P* < 0.01; ^*$$$$*^*P* < 0.0001). For western blot quantification, the Holm-Šídák method was used to compare groups (^*$$*^*P* < 0.01; ^*$$$*^*P* < 0.001; ^*$$$$*^*P* < 0.0001). Scale bars, 200 µm

To test whether the pathological synergy of *Tsc1* and *Depdc5* mutations was due solely to mTORC1 hyperactivation, we injected DKO mice with rapamycin, a chemical inhibitor of mTORC1. Interestingly, during the course of rapamycin administration, DKO mice resumed normal growth (Fig. 4a). After 10 days of rapamycin administration, liver/body weight ratios (Fig. 4b), as well as all serum markers of liver damage and dysfunction (Fig. 4c), showed dramatic recovery, indicating that mTORC1 hyperactivation is indeed the major cause of liver pathologies observed in DKO mice. Further confirming these observations, liver histology (Fig. 4d) and immunoblotting (Fig. 4d–f) indicated that 10 days of rapamycin administration was sufficient to rescue all examined liver pathologies, including mTORC1 hyperactivation (Fig. 4e), liver injury, inflammation and fibrosis (Fig. 4d–f).

DKO liver pathologies are associated with elevated PCNA staining (Supplementary Fig. S2d), which reflects regenerative responses to injury and damage. Rapamycin did not further elevate the PCNA staining intensity (Supplementary Fig. S2d), indicating that it relieves DKO pathologies mainly by restoring hepatocellular homeostasis, but not by promoting liver regeneration.

### Relieving ER stress unexpectedly aggravated DKO liver pathologies

**Fig. 5:**
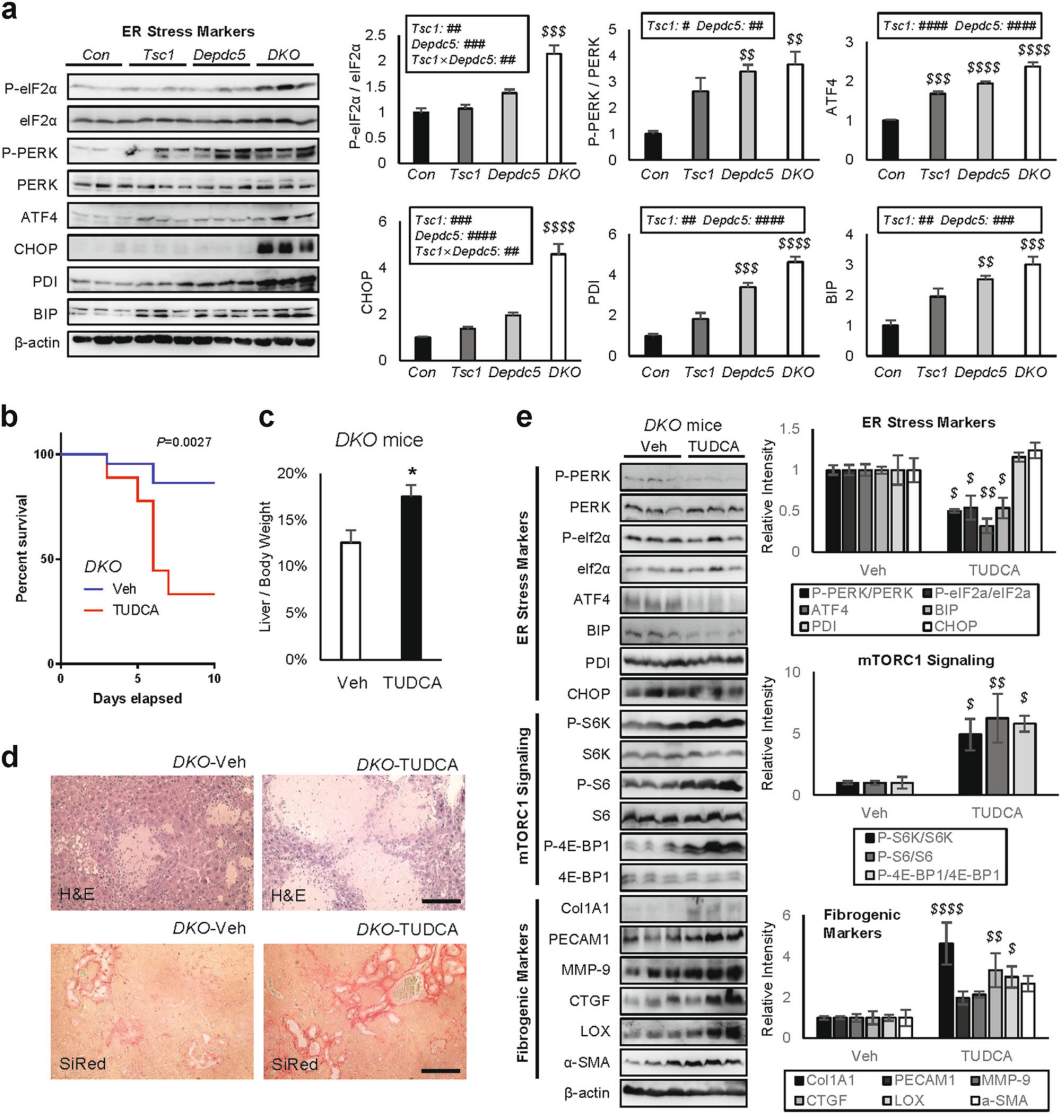
Relieving ER stress unexpectedly aggravated DKO liver pathologies. Mouse cohort described in Fig. 2 was subjected to immunoblotting **a**. Six-week-old DKO mice were injected daily with vehicle (Veh) or 500 mg/Kg TUDCA for 10 days (**b**–**e**; *n* ≥ 9). For drug treatment experiments, mice were gender-matched with both males and females. **a** ER stress signaling markers were examined from the indicated liver lysates through immunoblotting (left panels) and quantification (right panels). **b** Mouse survival was monitored throughout the course of the experiment. The *P* value was calculated through a log-rank test. **c** Liver/body weight ratio was measured at the experimental endpoint. **d** Liver sections were analyzed through H&E and SiRed staining. **e** Liver lysates were subjected to immunoblotting (left panels) and quantification (right panels) to examine ER stress signaling (top), mTORC1 signaling (middle) and fibrogenic markers (bottom). Data are presented as mean ± SEM (*n* ≥ 3) or actual values **b**. *P < 0.05 (Student’s *t*-test). Effects of *Tsc1* and *Depdc5* mutations and their interaction (*Tsc1* × *Depdc5*) were assessed through two-way ANOVA (^#^*P* < 0.05; ^##^*P* < 0.01; ^###^*P* < 0.001; ^####^*P* < 0.0001), and statistical significance between *Con* and indicated groups were assessed through Tukey’s multiple comparison test (^*$$*^*P* < 0.01; ^*$$$*^*P* < 0.001; ^*$$$$*^*P* < 0.0001). For western blot quantification, the Holm-Šídák method was used to compare groups (^*$*^*P* < 0.05; ^*$$*^*P* < 0.01; ^*$$$$*^*P* < 0.0001). Scale bars, 200 µm

Upregulated mTORC1 is known to increase ER stress^23^. Consistent with this, DKO livers exhibited prominent ER stress marker activation (Fig. 5a and Supplementary Fig. S2e), significantly stronger than *Tsc1* or *Depdc5* single knockouts (Fig. 5a). ER stress marker activation was strongly suppressed by rapamycin treatment (Supplementary Fig. S2f, g), indicating that mTORC1 hyperactivation in DKO livers provokes ER stress.

ER stress can be mitigated using chemical chaperones, such as tauroursodeoxycholic acid (TUDCA), which facilitates nascent protein folding in vivo^24,25^. Therefore, we injected DKO mice with TUDCA to relieve ER stress. Unexpectedly, more than half of the mice died during the 10 days of TUDCA administration (Fig. 5b). This was surprising because previous work showed that TUDCA injection was beneficial for WT mice, not lethal^24,25^. In addition, the surviving mice exhibited even greater liver/body weight ratios (Fig. 5c) and more severe liver histopathology associated with increased area of necrotic and fibrotic lesions (Fig. 5d).

In light of these observations, we questioned if TUDCA actually relieved ER stress in DKO mice. Immunoblotting showed that despite aggravated phenotypes, TUDCA generally reduced hepatic ER stress (Fig. 5e, top). Many ER stress markers, p-PERK, p-eIF2α, ATF4 and BIP, were significantly downregulated after TUDCA treatment; however, some markers, PDI and CHOP, did not change. Interestingly, mTORC1 signaling markers were all upregulated after TUDCA treatment (Fig. 5e, middle), suggesting that the presence of ER stress signaling limited mTORC1 activation. Expression of fibrogenic genes increased after TUDCA treatment (Fig. 5e, bottom), consistent with the observation that TUDCA and ER stress reduction actually worsened liver pathologies. These results indicate that ER stress is not a major conduit of DKO liver injury but may function as a negative feedback to limit mTORC1 activation.

### The DKO transcriptomic profile is distinct from those of control and single knockouts

**Fig. 6:**
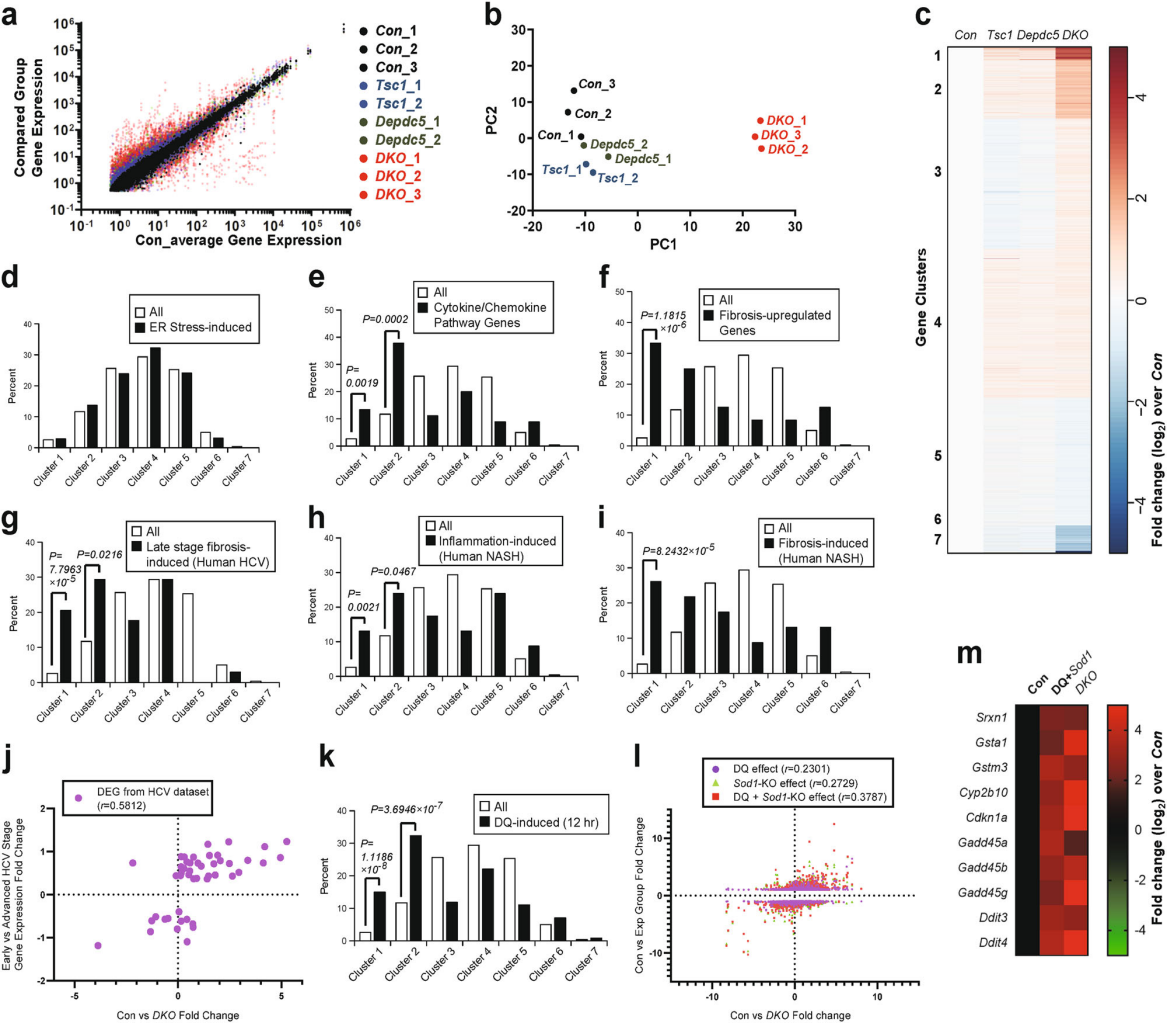
*Depdc5*/*Tsc1* double knockout (DKO) livers have distinct transcriptome profiles from single knockouts and specifically upregulate oxidative stress genes. Mouse cohort described in Fig. 2 was subjected to RNA-seq analyses. **a** Comparison of gene expression between averaged control profile and individual RNA-seq profiles from liver tissues of control (*n* = 3), *Tsc1*^*Δhep*^ (*n* = 2), *Depdc5*^*Δhep*^ (*n* = 2) and DKO (*n* = 3) mice. Each dot represents a single mRNA species in a dataset that is color-coded. For correlations between the datasets, see Supplementary Fig. S3a. **b** Principal component analysis (PCA) depicting the relationship between individual RNA-seq profiles. Each dot represents an entire RNA-seq profile from a single liver sample. Distinction is depicted spatially; similar profiles are clustered close together, while different profiles are located far from each other. **c** Transcript clusters were generated using a *k*-means algorithm (*n* = 7), using mRNA expression fold change values of *Tsc1*, *Depdc5*, and *DKO* samples over *Con* samples. **d**–**i**, **k** Bar graphs representing the distribution of all genes (white bars) and ER stress-inducible genes **d**, cytokine/chemokine pathway genes **e**, fibrosis-associated genes **f**, genes upregulated in late-stage fibrosis during human HCV infection **g**, genes upregulated in human NASH with lobular inflammation **h**, genes upregulated in human NASH with fibrosis **i** or genes induced in mouse liver at 12 h after diquat (DQ) treatment **k**. Gene enrichments in clusters 1 and 2 were examined by Fisher’s exact test, and *P* values were indicated on the graphs. **j**, **l** Comparison of gene expression fold changes induced in DKO livers (*X* axis in both panels) and HCV pathology progression **j** or DQ injection, *Sod1* mutation and both **l**. Correlations were assessed by computing a nonparametric Spearman correlation (*r*); *P* < 0.0001 for all correlation observations. **m** List of representative genes that are consistently upregulated by DQ + *Sod1* mutation and DKO mutation. Heat map diagrams represent mRNA expression fold change from averaged control values (expressed as log_2_ values)

Due to the unexpected results from ER stress suppression, we tried to approach pathogenetic mechanisms underlying DKO phenotypes more systematically. Therefore, we determined the transcriptomic profiles of livers from control, *Tsc1*^*Δhep*^, *Depdc5*^*Δhep*^, and DKO mice through RNA sequencing (Supplementary Table S1). Although *Tsc1*^*Δhep*^ and *Depdc5*^*Δhep*^ mice showed modest transcriptomic changes from control mice, DKO mice showed stronger deviations from the control liver transcriptomic profile (Fig. 6a). Heat map analysis of the correlations between individual datasets further demonstrated that DKO livers have a unique transcriptome profile that are most strongly correlated with each other, but not as strongly with controls or single knockouts (Supplementary Fig. S3a). Principal component analysis of all experimental replicates also indicated that *Con*, *Tsc1*^*Δhep*^ and *Depdc5*^*Δhep*^ samples exhibited relatively similar transcriptomic profiles, while DKO samples displayed a highly distinct profile (Fig. 6b). Likewise, although transcriptomic changes induced by single deletion of *Tsc1* or *Depdc5* correlated relatively well, DKO-induced changes had lower correlations with either single knockout-induced changes (Supplementary Fig. S3b). All of these results congruently indicate that DKO livers have transcriptomic profiles distinct from control or single knockout liver tissues.

### The DKO liver resembles diseased human livers with inflammation and fibrosis

To understand the nature of DKO-specific transcriptome differences, we classified the genes into 7 different categories through *k*-means clustering, according to their expression changes in *Tsc1*^*Δhep*^, *Depdc5*^*Δhep*^, and DKO livers (Fig. 6c and Supplementary Table S2). Among the 7 clusters, only a small number of genes were strongly and consistently upregulated, clusters 1 and 2, or downregulated, clusters 6 and 7, in DKO mice (Fig. 6c). Consistent with our immunoblotting findings (Fig. 5a), ER stress-responsive genes, such as *Atf4* and *Chop/Ddit3*, were most upregulated in DKO mice (Supplementary Fig. S3C). However, when we analyzed the whole known set of ER stress-inducible genes^26^, they were not overrepresented in clusters 1 and 2 (Fig. 6d), indicating that ER stress activation is not the major transcriptomic feature characterizing the DKO phenotype. This supports our pharmacological experiment showing that ER stress was not the conduit of liver pathology in DKO mice (Fig. 5).

In contrast to this, genes belonging to cytokine and chemokine signaling pathways were highly enriched in clusters 1 and 2 (Fig. 6e) and prominently upregulated in DKO livers (Supplementary Fig. S3d), indicating that inflammatory pathways characterize the DKO transcriptome. In addition, genes upregulated during tissue fibrosis, such as collagens, matrix metalloproteinases (MMPs), tissue inhibitors of metalloproteinase (TIMPs) and TGF-beta pathway genes, were also highly enriched in clusters 1 and 2 (Fig. 6f) and induced in DKO livers (Supplementary Fig. S3e). These are consistent with the extensive liver damage and fibrosis phenotypes we observed in the DKO mice.

Based on these observations, we were curious if the gene expression changes in DKO mouse livers had any resemblance to those induced by inflammation and fibrosis in human liver diseases. For this, we utilized recently published transcriptome profile datasets that were constructed using fibrotic human liver tissues associated with HCV infection^27^ or nonalcoholic steatohepatitis (NASH)^28^. Genes upregulated in late-stage fibrosis during HCV infection (Fig. 6g), lobular inflammation (Fig. 6h) and fibrosis (Fig. 6i) in NASH were highly enriched in clusters 1 and 2 (Fig. 6g–i). Since cluster 1 and 2 genes are strongly upregulated in the DKO mouse liver (Fig. 6c), these results indicate that DKO mouse liver models human inflammatory and fibrotic liver diseases associated with HCV and NASH. Furthermore, the gene expression changes associated with HCV fibrosis progression showed positive correlation with the changes induced by DKO (Fig. 6j). These results collectively indicate that DKO mice experience severe liver inflammation and fibrosis, transcriptomically similar to those associated with human HCV and NASH pathologies.

### Oxidative damage response pathways were upregulated in the DKO transcriptome

Inspection of clusters 1 and 2 identified that, in addition to the upregulated inflammation and fibrosis genes (Supplementary Fig. S3d, e), oxidative stress (Supplementary Fig. S3f) and DNA damage (Supplementary Fig. S3g) response genes were strongly upregulated in DKO mice. Sestrins (*Sesn1-3*) and Redds (*Ddit4* and *Ddit4l*), which are stress-inducible negative feedback regulators of the mTORC1 pathway^10,29^, were also upregulated in DKO livers (Supplementary Fig. S3h). Induction of Sestrin2 was detected at the protein level (Supplementary Fig. S2h), and activation of AMPK, a downstream target of Sestrin2, was also observed in DKO livers (Supplementary Fig. S2h). In contrast, major urinary proteins (Supplementary Fig. S3i) and cytochrome P450s (Supplementary Fig. S3j), whose expression is reduced during decreased growth hormone signaling^30^ or upon inflammation and oxidative stress^31–33^, respectively, were strongly downregulated in DKO mouse liver (Supplementary Fig. S3k, l). Although many of the cytochrome P450 genes were downregulated, some genes, such as *Cyp2b10* that is upregulated during hepatic damage and fibrosis^34,35^, were upregulated (Supplementary Fig. S3j) and found in cluster 1 (Supplementary Fig. S3l). These transcriptomic features indicate that DKO mouse livers specifically upregulate pathways responding to oxidative stress and subsequent DNA damage.

### DKO mouse liver exhibits excessive accumulation of superoxide radicals

**Fig. 7:**
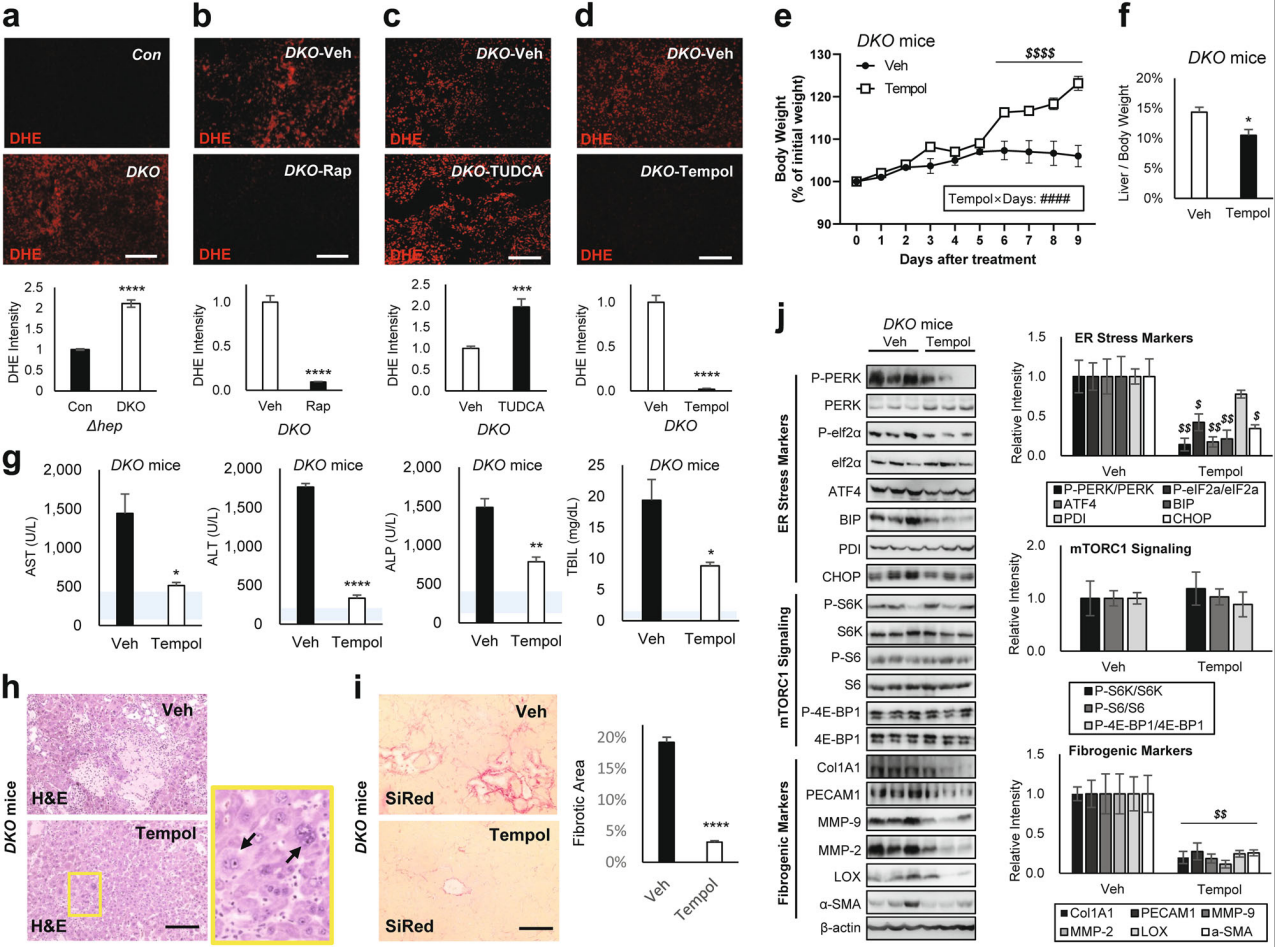
Superoxide dismutase mimetic Tempol corrects DKO liver pathologies. Mouse cohorts described in Figs. 2, 4 and 5 were analyzed. Littermate cohorts of six-week-old DKO mice were kept on vehicle drinking water (Veh) or 0.064% Tempol-containing water for 10 days (*n* ≥ 6). For drug treatment experiments, mice were gender-matched with both males and females. **a**–**d** Dihydroethidium (DHE) staining of liver sections and quantification. **e** Body weight was monitored throughout the course of the experiment. **f** Liver/body weight ratio was measured at the experimental endpoint. **g** Serum markers for liver damage were analyzed. Blue shaded regions indicate clinically normal ranges. **h** Liver sections were analyzed by H&E staining. Boxed area is magnified in right panel. **i** Liver sections were analyzed by SiRed staining. Fibrotic areas were quantified. **j** Liver lysates were subjected to immunoblotting (left panels) and quantification (right panels) to examine ER stress markers (top), mTORC1 signaling (middle), and fibrogenic markers (bottom). Data are presented as mean ± SEM (*n* = 3). *P < 0.05; **P < 0.01; ***P < 0.001; ****P < 0.0001 (Student’s *t*-test). Interaction between Tempol and treatment days (Tempol × Days) were assessed through RM two-way ANOVA (^####^*P* < 0.0001), and differences in individual data points were assessed through Sidak’s multiple comparison test (^*$$$$*^*P* < 0.0001). For western blot quantification, the Holm–Šídák method was used to compare groups (^*$*^*P* < 0.05; ^*$$*^*P* < 0.01). Scale bars, 200 µm

Upregulation of oxidative stress response genes implicates the presence of oxidative stress. Oxidative damage can precipitate a plethora of liver pathologies through DNA damage, inflammation, fibrosis, liver injury and hepatocyte death^36^, which were all observed from the DKO mouse liver. Therefore, we measured the level of hepatic oxidative stress by dihydroethidium (DHE) staining which visualizes superoxide radicals^37^. DKO livers had pronounced elevation of DHE staining intensity (Fig. 7a), which was blunted by rapamycin treatment (Fig. 7b). Interestingly, DHE intensity became more upregulated when DKO was treated with TUDCA (Fig. 7c), consistent with upregulation of mTORC1 and aggravation of liver pathologies (Fig. 5). These results indicate that DKO livers suffer severe oxidative stress with excessive accumulation of superoxide radicals.

### Superoxide insults produce transcriptomic changes similar to those of DKO

Superoxides can be formed by toxic chemicals such as diquat (DQ). Endogenous superoxide dismutase (*Sod1*) is important for reducing superoxides and suppressing their toxic effects^38^. Genes whose hepatic expression is induced by DQ treatment^38^ were highly enriched in clusters 1 and 2 (Fig. 6k), suggesting that DKO livers upregulated DQ-induced genes. In addition, the gene expression changes induced by DQ treatment, *Sod1* mutation, or both showed a positive correlation at the whole transcriptome level with DKO-induced changes (Fig. 6l). Accordingly, most of the DQ- and *Sod1* mutation-induced genes were also upregulated in DKO livers, and these genes included those involved in oxidative stress response, DNA damage response and ER stress (Fig. 6m and Supplementary Fig. S3). Taken together, we hypothesized that oxidative stress, especially the accumulation of superoxides, was one of the most characteristic features of DKO mouse livers.

### Superoxide radicals mediate liver pathologies induced by hyperactive mTORC1

To test whether the superoxide accumulation is the pathological conduit of DKO-induced mTORC1 hyperactivation, we treated the mice with Tempol, a membrane-permeable superoxide dismutase mimetic^39,40^. As expected, Tempol was highly effective in reducing DHE staining in DKO liver (Fig. 7d). Interestingly, Tempol-treated DKO mice exhibited significant weight gain after 5 days of treatment (Fig. 7e), indicating that like rapamycin, Tempol was able to release the DKO mice from systemic growth suppression. Furthermore, 10 days of Tempol administration was sufficient to reduce liver/body weight ratio (Fig. 7f), as well as serum markers for liver damage (Fig. 7g). Tempol also substantially reduced necrotic (Fig. 7h) and fibrotic (Fig. 7i) lesions exhibited by the DKO mouse liver (Fig. 7h, i). These observations were supported through western blot analyses, where Tempol treatment strongly reduced fibrotic marker expression in DKO mice (Fig. 7j). Interestingly, ER stress marker expression was also decreased by Tempol, indicating that superoxide accumulation also contributed to the mTORC1-induced ER stress (Fig. 7j). However, phosphorylation of mTORC1 downstream targets was not suppressed by Tempol, confirming that Tempol specifically reduced superoxide accumulation without affecting mTORC1 signaling (Fig. 7j). Consistent with the observation that mTORC1 is still hyperactivated, Tempol administration did not suppress hepatocyte hypertrophy (Fig. 7h, arrows), while other pathological features were substantially suppressed (Fig. 7e–j).

Suppression of liver pathologies was again observed when DKO mice were treated with N-acetylcysteine (NAC), another antioxidant that scavenges superoxide radicals (Supplementary Fig. S4). Collectively, these results indicate that production of reactive oxygen species, such as superoxide radicals, is the major pathological conduit of how hyperactive mTORC1 in DKO mice induces liver injury and precipitates pathologies.

### DKO mice have defective glucose metabolism and hepatic insulin resistance

**Fig. 8:**
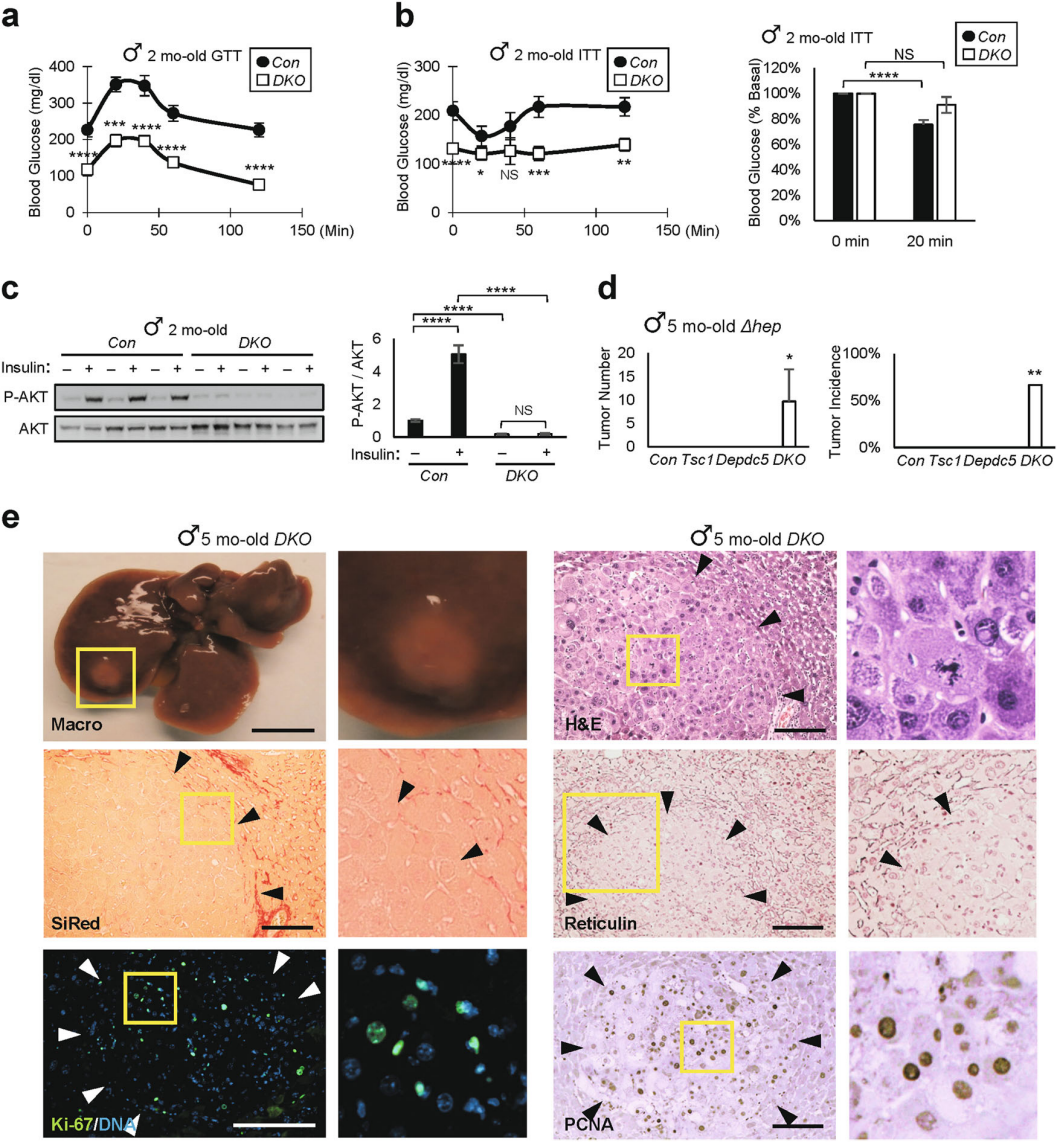
*Depdc5*/*Tsc1* double knockout mice exhibit insulin resistance and hepatocellular carcinoma. **a**–**c** After 4–6 h of fasting, Two-month-old Con and DKO littermates (*n* ≥ 9) were subjected to **a** glucose and **b** insulin tolerance tests (GTT and ITT, respectively). Data are normalized according to baseline glucose levels (**b**, right). **c** Livers were collected from Con and DKO littermates, after 4 h of fasting, before (–) or 5 min after ( + ) an insulin injection, homogenized and analyzed through immunoblotting (left) and quantification (right; *n* = 6). **d** Tumor number and incidence of the indicated five-month-old mice (*n* ≥ 3). All mouse strains except DKO were free of liver tumor. **e** Macroscopic images (Macro) and histology images of H&E, SiRed, Reticulin, Ki-67 and PCNA staining were shown for five-month-old DKO mouse livers. Arrowheads indicate approximate boundaries of tumor nodules. Boxed areas are magnified in right panels. Data are presented as mean ± SEM. *P < 0.05, **P < 0.01, ***P < 0.001, ****P < 0.0001, NS (*P* = 0.0712, 0.1799 and 0.6796 in **b**-left, **b***-*right and **c**, respectively) from a Student’s *t*-test. Scale bars, 200 µm (histology) and 1 cm (whole liver)

DKO mice experienced hypoglycemia (Fig. 8a and Supplementary Fig. S5a), likely due to hepatic dysfunction and subsequent reduction in hepatic glucose output. Blood glucose levels of DKO mice were not reduced in response to insulin (Fig. 8b and Supplementary Fig. S5b, c), and DKO hepatocytes in intact livers did not activate AKT in response to insulin stimulation (Fig. 8c). One potential explanation for this is due to mTORC1 and ER stress hyperactivation, both of which are known to provoke insulin resistance by blocking the insulin receptor-AKT pathway^24,41^.

### DKO mice develop hepatocellular carcinoma

Although *Depdc5*^*Δhep*^ or *Tsc1*^*Δhep*^ mice developed mild inflammation at 5-6 months (Fig. 1)^18^, they did not exhibit liver cancer until they reached 9–15 months^17,18^. Since DKO mice experienced more pronounced liver damage at a much earlier age, we hypothesized that DKO livers would more quickly progress to liver cancer. Indeed, five-month-old mice revealed macroscopically visible liver tumors (Fig. 8d, e, Macro). More tumor nodules were discovered in histological sections (Fig. 8e, H&E, and Supplementary Fig. S5d), which frequently displayed atypical mitotic features (Fig. 8e, H&E right panel). The nodules were surrounded by fibrotic tissue, but the nodules themselves were devoid of fibrosis (Fig. 8e, SiRed, and Supplementary Fig. S5e). Most of these nodules also displayed markedly decreased reticulin staining (Fig. 8e, Reticulin, and Supplementary Fig. S5f) and elevated frequency of Ki-67 and PCNA staining (Fig. 8e, Ki-67 and PCNA), indicating that they are indeed hepatocellular carcinoma.

## Discussion

mTORC1 is a protein kinase important for liver metabolism and is regulated by two small GTPases, Rheb and Rag^1–4^. Rheb mediates growth factor regulation, while Rag mediates stress and nutrient control. Although the Rheb and Rag pathways were extensively studied, there have been no genetic studies of whether these two pathways interact for physiological mTORC1 regulation in an intact multicellular organism.

Rheb and Rag are regulated by their respective GAPs, TSC and GATOR1^4,5^. TSC1 and DEPDC5 are essential components of TSC and GATOR1, respectively. *Tsc1* deletion in mouse liver produced pleiotropic metabolic phenotypes such as suppression of fat oxidation^42^ and ketogenesis^43^, increased FGF21 production^44^, and decreased insulin sensitivity and lipogenesis^22^. However, *Tsc1* deletion in the liver (*Tsc1*^*Δhep*^) did not cause gross pathologies in young mice, although it promoted age-associated liver inflammation and carcinogenesis in one-year-old mice^17,18^. A physiological role for Depdc5 in the liver was not formerly investigated until the current study. Here, we showed that *Depdc5* deletion in mouse liver upregulated hepatic mTORC1 most prominently in zone 3, where oxygen and nutrients are relatively scarce. Since GATOR1 is important for suppressing mTORC1 in nutrient-depleted conditions^8^, it is plausible that GATOR1 is critical for regulating mTORC1 in zone 3 hepatocytes. After maturation and aging of *Depdc5*^*Δhep*^ mice, mTORC1 upregulation became more pronounced and resulted in phenotypes similar to *Tsc1*^*Δhep*^ mice, such as mild inflammation and decreased fat levels. Consistent with their similar mild phenotypes, *Depdc5*^*Δhep*^ and *Tsc1*^*Δhep*^ mice had similar transcriptomic profiles that were only moderately different from the wild-type profile.

By crossing *Depdc5*^*Δhep*^ mice with *Tsc1*^*Δhep*^ mice, we showed that mutations in both *Depdc5* and *Tsc1* generate a synergistic genetic interaction and produce a very strong hyperactivation of mTORC1. This provides genetic evidence in animal models confirming that the Rheb and Rag pathways indeed interact for mTORC1 regulation in a physiological context. mTORC1 hyperactivation in DKO mice resulted in liver dysfunction associated with prominent hepatocyte injury and fibrosis by two-months of age. This led to dramatic elevation of liver damage markers in the serum. Excessive bilirubin accumulation in serum led to an externally observable jaundice phenotype. In addition, since the liver is the primary source of insulin-like growth factors that are essential for systemic growth, liver failure in DKO mice also suppressed growth. At the liver transcriptome level, specific stress response pathways, such as oxidative stress, inflammation, DNA damage and cell death pathways were strongly upregulated. All of these striking phenotypes were not manifested in either *Tsc1*^*Δhep*^ or *Depdc5*^*Δhep*^ single knockout strains or in any formerly described models of mTORC1 activation, such as *Deptor* knockout mice^45^. Therefore, our current work provides a unique model of unregulated mTORC1 activation and shows that mTORC1 hyperactivation by itself can disrupt hepatocellular homeostasis, provoking liver injury and failure.

mTORC1 is regulated through multiple negative feedback loops. mTORC1 hyperactivation is known to inhibit Akt through S6K- or Grb10-mediated feedback inhibition of insulin signaling^41,46,47^. Since Akt is an mTORC1 upregulator, Akt inhibition can limit mTORC1 activation. However, at the same time, inhibition of insulin-AKT signaling can also precipitate metabolic insulin resistance. Correspondingly, DKO mouse livers exhibited strong insulin resistance, and hepatocytes from DKO mice did not activate AKT in response to insulin. Although the DKO liver suffers strong insulin resistance, the blood glucose level was rather strongly decreased due to the deterioration of hepatocyte homeostasis and subsequent reduction in hepatic glucose output.

In addition to the feedback loop involving insulin signaling, Sestrins can also provide a negative feedback mechanism for the mTORC1 pathway. In Drosophila, Sestrin is an important feedback inhibitor of the mTORC1 pathway through Tsc1/2^48^ and Depdc5^49^ pathways. In the current work, we found that Sestrins expression levels were substantially elevated after deletion of Tsc1, Depdc5 or both. AMPK, one of the downstream effectors of Sestrins inhibiting mTORC1^10^, was subsequently activated in these tissues. Redd1 (*Ddit4*) and Redd2 (*Ddit4l*), which inhibit mTORC1 through Tsc1/Tsc2 upregulation^29^, were also upregulated in mTORC1-activated liver tissues. Therefore, it is possible that, in our mTORC1 activation models, Sestrins and Redds may have resulted in negative feedback inhibition to limit mTORC1 activities.

It was quite striking that all of the liver pathologies in DKO mice were almost completely rescued by only 10 days of rapamycin treatment. Liver/body weight ratios were restored to normal levels, and liver damage markers in the serum also recovered close to clinically normal ranges. Although rapamycin was historically considered a growth attenuator, rapamycin-mediated normalization of liver homeostasis actually promoted systemic growth in this specific DKO model. Upon rapamycin treatment, necrotic and fibrotic lesions in DKO mice disappeared, and hepatocellular ER stress, oxidative stress and apoptosis were all relieved. Therefore, mTORC1 is indeed the major conduit of how the Rheb and Rag pathways pathogenetically interact to produce liver injury and failure.

mTORC1 upregulation increases protein synthesis, which can put a burden on protein folding machinery and therefore induce accumulation of unfolded proteins in the ER, also known as ER stress^23^. DKO mouse livers exhibited upregulation of ER stress signaling at the protein level, confirming that mTORC1 hyperactivation in DKO mice indeed precipitated unfolded protein accumulation and ER stress. However, the ER stress response pathway was not overrepresented in the DKO transcriptome, raising questions of whether the ER stress pathway is important for DKO pathologies. Indeed, TUDCA, a chemical chaperone that effectively suppressed hepatocellular ER stress in DKO mouse liver, was completely ineffective in rescuing DKO liver pathologies. Instead, TUDCA-treated DKO mice increased mTORC1 activation, further potentiating liver pathologies in DKO mice to the point of fatality. Even in the surviving mice, TUDCA administration increased expression of fibrogenic markers and more extensively damaged hepatocytes. It is possible that ER stress signaling somehow limits mTORC1 activation, reducing its negative consequences on liver health. These data also indicate that ER stress signaling is not the major mechanism of how hyperactive mTORC1 disrupts hepatocellular homeostasis.

In addition to inducing ER stress, mTORC1 hyperactivation can elevate oxidative stress by altering mitochondrial metabolism^50,51^, inhibiting autophagic elimination of dysfunctional mitochondria^48,52^, and suppressing the superoxide-scavenging action of Sod1^53^. Indeed, DKO livers experienced severe oxidative stress associated with excessive accumulation of superoxide radicals and exhibited a transcriptomic profile that is similar to DQ-induced oxidative stress and *Sod1* loss. This high level of oxidative stress can damage cellular macromolecules including DNA. Consistent with this, the DKO transcriptome also exhibited upregulation of some DNA damage response genes. Administration of chemical antioxidants that scavenge superoxide radicals, such as Tempol and NAC, effectively reduced hepatic oxidative stress. Surprisingly, 10 days of antioxidant administration was sufficient to normalize almost every liver pathology parameter observed in DKO mouse liver and even restored normal growth. Since mTORC1 signaling itself was not suppressed by chemical antioxidants, these results indicate that hyperactive mTORC1 signaling provokes liver failure primarily through the induction of superoxide radicals that injure hepatocytes.

At the tissue level, mTORC1 hyperactivation produced crosstalk with a number of additional pathways. For instance, NF-kB target genes such as *Il6*^54^ and *Cd44*^55^, TGF-beta signaling targets genes *Acta2*, *Mmp2* and *Timp2*^56^ and Hippo-Yap target genes *Ctgf*^57^ and *Notch2*^58^, were all upregulated in DKO mice. These signaling pathways were implicated in inflammation-dependent acceleration of carcinogenesis in previous studies^59^. Consistent with the finding and former studies, we found that the DKO mice spontaneously developed HCC at 5 months, a relatively early age.

Our observations also provide an explanation of how human genetic variations in the *DEPDC5* gene can accelerate HBV/HCV-associated liver pathologies such as hepatic fibrosis^14^ and carcinogenesis^13,15^. HBV and HCV infections upregulate mTORC1 by activating PI3K-AKT signaling and/or inhibiting TSC, both of which subsequently activate Rheb^60,61^. Genetic variations suppressing DEPDC5 function would upregulate Rag signaling, and this would synergistically interact with the HBV/HCV infection that elevates Rheb signaling. Concomitant upregulation of both Rag and Rheb axes would lead to mTORC1 hyperactivation that can precipitate oxidative liver pathologies, as observed in the DKO mice described here. Furthermore, we found that our DKO liver transcriptome is closely related with human HCV and NASH transcriptomes. Therefore, our DKO mice provide a novel mouse model for investigating the role of human *DEPDC5* variations in accelerating liver pathologies associated with HCV and NASH. However, the DKO model currently described here does not involve an actual viral infection or virus-associated activation of adaptive immunity. Therefore, additional studies should be conducted in the context of actual HBV and HCV infection to gain a more direct translation of our findings into the corresponding human liver pathologies.

In conclusion, we show that the Rag and Rheb pathways are both required for maximum mTORC1 activation in tissues. Correspondingly, double knockout of the *Tsc1* and *Depdc5* genes provokes prominent upregulation of mTORC1, disrupts hepatocellular homeostasis, and subsequently precipitates oxidative injury and subsequent liver failure. Our work provides a valuable model for examining the consequences of mTORC1 hyperactivation, understanding human liver pathologies associated with HCV, NASH and *DEPDC5* variation, and developing therapeutic strategies for treating such pathologies with mTORC1 inhibitors or antioxidant compounds.

## Materials and methods

### Mice and diet

*Depdc5*^*F/F*^ (EM: 10459) mice, originated from the HEPD0734_3_G10 embryonic stem cell clone, were obtained from the European Mouse Mutant Archive. *Depdc5*^*F/F*^ mice were bred to *Albumin* (*Alb*)*-Cre* to produce hepatocyte-specific knockout mice. DKO mice were generated by interbreeding *Depdc5*^*F/F*^ and *Tsc1*^*F/F*^ mice^17,62^, then breeding progeny with *Alb-Cre* mice. *Depdc5* single knockout experiments were done in C57BL/6 background. *Tsc1* mice were originally produced in a 129S4/SvJae background^17,62^ but were backcrossed to C57BL/6 for more than three generations for DKO experiments. To minimize genetic and environmental variations, littermate controls were used throughout the study, and mice were cohoused. For instance, *Depdc5*^*Δhep*^ (*Alb-Cre*/*Depdc5*^*F/F*^) and *Depdc5*^*F/F*^ littermates were used for *Depdc5* single knockout experiments. *Alb-Cre*/*Tsc1*^*F/+*^/*Depdc5*^*F/+*^ and *Tsc1*^*F/F*^/*Depdc5*^*F/F*^ breeders produced control (*Alb-Cre* negative mice and *Alb-Cre*/*Tsc1*^*F/+*^/*Depdc5*^*F/+*^ mice), *Tsc1*^*Δhep*^ (*Alb-Cre*/*Tsc1*^*F/F*^/*Depdc5*^*F/+*^), *Depdc5*^*Δhep*^ (*Alb-Cre*/*Tsc1*^*F/+*^/*Depdc5*^*F/F*^), and *Tsc1*^*Δhep*^/*Depdc5*^*Δhep*^ (DKO, *Alb-Cre*/*Tsc1*^*F/F*^/*Depdc5*^*F/F*^) littermates that were analyzed for genetic interaction assays. *Alb-Cre*/*Tsc1*^*F/F*^/*Depdc5*^*F/F*^ males and *Tsc1*^*F/F*^/*Depdc5*^*F/F*^ females produced DKO littermate cohorts for drug intervention experiments. Mice were maintained in filter-topped cages with cob bedding and given free access to autoclaved regular chow/low fat diet (LFD, Lab Diet 5L0D), high fat diet (HFD, Bio-Serv S3282), and water, as previously described^63^. When indicated, freshly made rapamycin (10 mg/Kg body weight), tauroursodeoxycholic acid (TUDCA, 500 mg/Kg body weight), N-acetylcysteine (NAC, 250 mg/Kg body weight) or vehicle (5% Tween 80, 5% PEG400; or PBS) solutions were administered once daily through intraperitoneal (i.p.) injections for the last 10 days. A superoxide dismutase mimetic 4-hydroxy-2,2,6,6-tetramethylpiperidin-1-oxyl (Tempol, 0.064%) was administered to mice through drinking water. Acetaminophen (APAP, 400 mg/Kg body weight) was administered through a single i.p. injection after 12 h of fasting. Glucose (1 g/Kg glucose) and insulin (0.65 U/Kg insulin) tolerance tests (GTT/ITT) were done according to previously described procedures^25^. For acute insulin response studies, mice were put under a surgical plane of isoflurane anesthesia. First, one part of the liver was collected as an untreated control. Then 0.8 U/Kg insulin, diluted in PBS, was injected intravenously through the vena cava. After 5 min, the other parts of the liver were collected as an insulin-treated sample. Information regarding mouse number, age, gender, diet duration, drug dose, route and frequency are indicated in the corresponding Figure and Figure legends. All animal procedures were ethically approved by the Institutional Animal Care & Use Committee and overseen by the Unit for Laboratory Animal Medicine at the University of Michigan.

### Antibodies and reagents

Antibodies for DEPDC5 were generated from Pocono Rabbit Farm & Laboratory using bacterially expressed recombinant proteins. We obtained COL1A1 (sc-293182), Pro-COL3A1 (sc-166316), PECAM-1 (sc-376764), MMP-2 (sc-53630), MMP-3 (sc-21732), MMP-9 (sc-393859), LOX (sc-373995), MMP-13 (sc-515284), CTGF (sc-365970), S6K (sc-230), eIF2α (sc-11386), ATF4 (sc-200 and sc-22800), and TIMP-3 (sc-373839) antibodies from Santa Cruz Biotechnology, Actin (9E10) antibody from Developmental Studies Hybridoma Bank, phospho-Thr389-S6K (9234), pThr172-AMPK (2535), pThr37/46-4E-BP (2855), 4E-BP (9452), pSer51-eIF2α (3398), pThr980-PERK (3179), PERK (5683), PDI (3501), BIP (3177), CHOP (2895), pSer473-AKT (4060), AKT (4691), pSer236/239-S6 (2211) and S6 (2317) from Cell Signaling Technology, α-smooth muscle actin (α-SMA, ab5694) antibody from Abcam, and F4/80 (MF48000) antibody from Invitrogen. Acetaminophen, NAC and Tempol are from Sigma, TUDCA is from Cayman Chemical, and rapamycin is from LC labs.

### Histology

Liver tissues were fixed in 10% buffered formalin, embedded in paraffin and subjected to hematoxylin and eosin (H&E) staining and immunohistochemical staining, as previously described^63^. In brief, paraffin-embedded liver sections were incubated with primary antibody (1:100), followed by incubation with biotin-conjugated secondary antibodies (Vector Lab, BA-9200 or BA-9401; 1:200) and horseradish peroxidase (HRP)-conjugated streptavidin (BD Biosciences, 554066; 1:300). The HRP activity was visualized with diaminobenzidine staining. Hematoxylin counterstaining was applied to visualize nuclei. For α-SMA and Ki-67 staining, Alexa Flour 488 or 594-conjugated secondary antibodies (Invitrogen) were used to visualize primary antibody staining. Terminal deoxynucleotidyl transferase dUTP Nick-End Labeling (TUNEL) assays were performed using In Situ Cell Death Detection Kit-TMR-Red (Roche). Dihydroethidium (DHE) staining was performed using freshly frozen liver sections and DHE (Thermo Fisher Scientific, D11347) as formerly described^25^. To visualize collagen fibers, liver sections were stained with saturated picric acid containing 0.1% Sirius Red (SiRed, Sigma). For Oil Red O staining, OCT-embedded frozen liver sections were dried and stained with fresh 0.5% Oil Red O solution for 15 min then rinsed with 60% isopropanol. Reticulin staining was performed using a kit from Polyscience (25094), following the manufacturer’s recommendation. Histology samples were analyzed under an epifluorescence-equipped light microscope from Meiji.

### Immunoblotting

Cells and tissues were lysed in radioimmunoprecipitation assay (RIPA) buffer (50 mM Tris–HCl, pH 7.4; 150 mM NaCl; 1% sodium deoxycholate; 1% NP-40; 1% Triton X-100; and complete protease inhibitor cocktail (Roche)). Lysates were clarified by centrifugation, and protein concentration was normalized using Bio-rad protein assay dye reagent. Protein lysates were boiled in SDS sample buffer for 5 min, separated by SDS-PAGE, transferred to PVDF membranes and subjected to immunoblotting procedures. 5% blocking grade non-fat milk (170–6404 from Bio-Rad) in TBST was used for membrane blocking and antibody incubation. 1X western blocking reagent (11 921 673 001 from Roche) in TBST was used for phospho-specific primary antibody incubation. Primary antibodies from Santa Cruz Biotechnology and Developmental Studies Hybridoma Bank were used at 1:100, and all the other primary antibodies were used at 1:1000. HRP-conjugated secondary antibodies were purchased from Bio-Rad and used at 1:2000. Chemiluminescence was detected using LAS4000 (GE) systems.

### Serum chemistry

Blood was obtained by cardiac puncture and separated by centrifugation to obtain serum. Serum chemistry markers associated with liver cytotoxicity (ALT, alanine aminotransaminase; AST, aspartate aminotransferase) or liver function (ALP, alkaline phosphatase; TBIL, total bilirubin) were obtained through standard operating procedures using the Liasys clinical chemistry system (AMS Alliance) within the In Vivo Animal Core of the Unit for Laboratory Animal Medicine.

### RNA-Seq data analysis

In total 10 µg of DNAase I-treated total RNA, purified from liver tissues of control (*Tsc1*^*F/F*^/*Depdc5*^*F/F*^; *n* = 3), *Tsc1*^*Δhep*^ (*Alb-Cre*/*Tsc1*^*F/F*^; *n* = 2), *Depdc5*^*Δhep*^ (*Alb-Cre*/*Depdc5*^*F/F*^; *n* = 2) and *DKO* (*Alb-Cre*/*Tsc1*^*F/F*^/*Depdc5*^*F/F*^; *n* = 3) mice, were submitted to BGI for mRNA enrichment, library construction and sequencing (BGISeq 50SE), and processed through standard experimental and analytical pipelines. Each sample produced more than 20 M clean reads, that were mapped to the mm9 reference genome using STAR^64^. Then, Cufflinks was used to generate Fragments Per Kilobase of transcript per Million mapped reads (FPKM) table^65^, supplied as Supplementary Table S1. Genes with >0.5 FPKM values in every dataset were used to perform correlation and *k*-means clustering analyses. The accession number for the RNA-seq data reported in this paper is GSE136684.

As formerly described^66^, gene enrichment analyses were performed to identify whether a subset of genes were significantly overrepresented in specific gene clusters. Inflammation and fibrosis upregulated gene lists were obtained from recent transcriptome data on human liver samples with HCV-associated fibrosis^27^ and nonalcoholic steatohepatitis (NASH)^28^. From the HCV dataset, disease progression-associated fold changes of differentially expressed genes (the most stringent set with study-wide significance; both up- and down-regulated genes) were compared with the *DKO*-induced gene expression fold changes. The oxidative stress-upregulated gene list was obtained from livers of mice acutely treated with Diquat (DQ) for 12 h^38^. From the same dataset, DQ-treated and *Sod1*-knockout induced fold changes of differentially expressed genes (both up- and down-regulated genes; 1 h DQ treatment) were compared with the *DKO*-induced gene expression fold changes. The ER stress-upregulated gene list was obtained from tunicamycin-treated mouse embryonic fibroblasts^26^. Cytokine and chemokine pathway gene lists were generated by selecting relevant genes from the list of genes whose names begin with *Ccl/Ccr*, *Cxcl*/*Cxcr*, *Il/Ilr*, *Ifn/Ifnr* and *Tnf/Tnfr*. The fibrosis-associated gene list was generated by selecting relevant genes from the list of genes whose names begin with *Col*, *Mmp*, *Timp* and *Tgfb*. Cytochrome P450 and major urinary protein gene lists were generated by selecting relevant genes from the list of genes whose names begin with *Cyp* and *Mup*, respectively.

### Quantification and statistics

Immunoblot images were quantified by densitometry, and protein expressions were expressed as relative band intensities. Histological images were analyzed by densitometric or fluorometric methods as appropriate. When indicated, data are shown as mean ± SEM. Statistical significance between two groups was calculated using a Student’s *t*-test (*P < 0.05; **P < 0.01; ***P < 0.001; ****P < 0.0001). When multiple parameters were assessed, the Holm–Šídák method was used to compare groups (^*$*^*P* < 0.05; ^*$$*^*P* < 0.01; ^*$$$*^*P* < 0.001; ^*$$$$*^*P* < 0.0001). A two-way ANOVA was used to evaluate the effect of *Tsc1* and *Depdc5* mutations and assess interactions and synergy between them (^#^*P* < 0.05; ^*##*^*P* < 0.01; ^###^*P* < 0.001; ^####^*P* < 0.0001), and statistical significance between two individual groups were assessed through Tukey’s multiple comparison test (^*$$*^*P* < 0.01; ^*$$$*^*P* < 0.001; ^*$$$$*^*P* < 0.0001). The effect of drugs on body weights was assessed through repeated measures (RM) 2-way ANOVA to evaluate the interaction between treatment and time (^####^*P* < 0.0001). Differences in individual data points were assessed through Sidak’s multiple comparison test (^*$*^*P* < 0.05; ^*$$*^*P* < 0.01; ^*$$$*^*P* < 0.001; ^*$$$$*^*P* < 0.0001). Survival curves were compared with a log-rank test. Statistical significance of gene enrichment in a specific cluster was calculated using Fisher’s Exact test. Correlations between RNA-seq datasets were assessed by computing nonparametric Spearman correlation (*r*); *P* < 0.0001 for all correlation observations. GraphPad Prism 8 was used for all statistical analyses except *k*-means clustering analyses and gene enrichment analyses, which were performed using R.

## Supplementary information

Supplementary Table S1

Supplementary Table S2

Supplementary Information

## Supplementary information

**Supplementary Information** accompanies the paper at (10.1038/s41421-019-0131-9).

## References

[1] Hay N, Sonenberg N (2004). Upstream and downstream of mTOR. Genes Dev..

[2] Wullschleger S, Loewith R, Hall MN (2006). TOR signaling in growth and metabolism. Cell.

[3] Zoncu R, Efeyan A, Sabatini DM (2011). mTOR: from growth signal integration to cancer, diabetes and ageing. Nat. Rev. Mol. Cell Biol..

[4] Gonzalez A, Hall MN (2017). Nutrient sensing and TOR signaling in yeast and mammals. EMBO J..

[5] Bar-Peled L, Sabatini DM (2014). Regulation of mTORC1 by amino acids. Trends Cell Biol..

[6] Inoki K, Li Y, Zhu T, Wu J, Guan KL (2002). TSC2 is phosphorylated and inhibited by Akt and suppresses mTOR signalling. Nat. Cell Biol..

[7] Inoki K, Zhu T, Guan KL (2003). TSC2 mediates cellular energy response to control cell growth and survival. Cell.

[8] Bar-Peled L (2013). A Tumor suppressor complex with GAP activity for the Rag GTPases that signal amino acid sufficiency to mTORC1. Science.

[9] Panchaud N, Peli-Gulli MP, De Virgilio C (2013). Amino acid deprivation inhibits TORC1 through a GTPase-activating protein complex for the Rag family GTPase Gtr1. Sci. Signal.

[10] Ho A, Cho CS, Namkoong S, Cho US, Lee JH (2016). Biochemical basis of sestrin physiological activities. Trends Biochem. Sci..

[11] Dibbens LM (2013). Mutations in DEPDC5 cause familial focal epilepsy with variable foci. Nat. Genet..

[12] Ishida S (2013). Mutations of DEPDC5 cause autosomal dominant focal epilepsies. Nat. Genet..

[13] Miki D (2011). Variation in the DEPDC5 locus is associated with progression to hepatocellular carcinoma in chronic hepatitis C virus carriers. Nat. Genet.

[14] Burza MA (2016). DEPDC5 variants increase fibrosis progression in Europeans with chronic hepatitis C virus infection. Hepatology.

[15] Liu W (2019). Correlation between the DEPDC5 rs1012068 polymorphism and the risk of HBV-related hepatocellular carcinoma. Clin. Res. Hepatol. Gastroenterol..

[16] Umemura A (2014). Liver damage, inflammation, and enhanced tumorigenesis after persistent mTORC1 inhibition. Cell Metab..

[17] Kenerson HL (2013). Akt and mTORC1 have different roles during liver tumorigenesis in mice. Gastroenterology.

[18] Menon S (2012). Chronic activation of mTOR complex 1 is sufficient to cause hepatocellular carcinoma in mice. Sci. Signal.

[19] Chen W (2017). Tethering interleukin-22 to apolipoprotein A-I ameliorates mice from acetaminophen-induced liver injury. Theranostics.

[20] Borude P (2018). Pleiotropic Role of p53 in injury and liver regeneration after acetaminophen overdose. Am. J. Pathol..

[21] Lee DH (2018). Inactivation of Sirtuin2 protects mice from acetaminophen-induced liver injury: possible involvement of ER stress and S6K1 activation. BMB Rep..

[22] Yecies JL (2011). Akt stimulates hepatic SREBP1c and lipogenesis through parallel mTORC1-dependent and independent pathways. Cell Metab..

[23] Ozcan U (2008). Loss of the tuberous sclerosis complex tumor suppressors triggers the unfolded protein response to regulate insulin signaling and apoptosis. Mol. Cell.

[24] Ozcan U (2006). Chemical chaperones reduce ER stress and restore glucose homeostasis in a mouse model of type 2 diabetes. Science.

[25] Park HW (2014). Hepatoprotective role of Sestrin2 against chronic ER stress. Nat. Commun..

[26] Han J (2013). ER-stress-induced transcriptional regulation increases protein synthesis leading to cell death. Nat. Cell Biol..

[27] Ramnath D (2018). Hepatic expression profiling identifies steatosis-independent and steatosis-driven advanced fibrosis genes. JCI Insight.

[28] Gerhard GS (2018). Transcriptomic profiling of obesity-related nonalcoholic steatohepatitis reveals a core set of fibrosis-specific genes. J. Endocr. Soc..

[29] Ellisen LW (2005). Growth control under stress: mTOR regulation through the REDD1-TSC pathway. Cell Cycle.

[30] Knopf JL, Gallagher JF, Held WA (1983). Differential, multihormonal regulation of the mouse major urinary protein gene family in the liver. Mol. Cell Biol..

[31] Morgan ET (2001). Regulation of cytochrome p450 by inflammatory mediators: why and how?. Drug Metab. Dispos..

[32] El-Kadi AO, Bleau AM, Dumont I, Maurice H, du Souich P (2000). Role of reactive oxygen intermediates in the decrease of hepatic cytochrome P450 activity by serum of humans and rabbits with an acute inflammatory reaction. Drug Metab. Dispos..

[33] Aitken AE, Richardson TA, Morgan ET (2006). Regulation of drug-metabolizing enzymes and transporters in inflammation. Annu Rev. Pharm. Toxicol..

[34] Shan W (2008). Peroxisome proliferator-activated receptor-beta/delta protects against chemically induced liver toxicity in mice. Hepatology.

[35] Koga T (2016). Regulation of cytochrome P450 2B10 (CYP2B10) expression in liver by peroxisome proliferator-activated receptor-beta/delta modulation of SP1 promoter occupancy. J. Biol. Chem..

[36] Parola M, Robino G (2001). Oxidative stress-related molecules and liver fibrosis. J. Hepatol..

[37] Zhao H (2003). Superoxide reacts with hydroethidine but forms a fluorescent product that is distinctly different from ethidium: potential implications in intracellular fluorescence detection of superoxide. Free Radic. Biol. Med..

[38] Han ES (2008). The in vivo gene expression signature of oxidative stress. Physiol. Genomics.

[39] Muscoli C (2003). On the selectivity of superoxide dismutase mimetics and its importance in pharmacological studies. Br. J. Pharm..

[40] Thiemermann C (2003). Membrane-permeable radical scavengers (tempol) for shock, ischemia-reperfusion injury, and inflammation. Crit. Care Med.

[41] Um SH, D’Alessio D, Thomas G (2006). Nutrient overload, insulin resistance, and ribosomal protein S6 kinase 1, S6K1. Cell Metab..

[42] Kucejova B (2016). Hepatic mTORC1 opposes impaired insulin action to control mitochondrial metabolism in obesity. Cell Rep..

[43] Sengupta S, Peterson TR, Laplante M, Oh S, Sabatini DM (2010). mTORC1 controls fasting-induced ketogenesis and its modulation by ageing. Nature.

[44] Cornu M (2014). Hepatic mTORC1 controls locomotor activity, body temperature, and lipid metabolism through FGF21. Proc. Natl Acad. Sci. USA.

[45] Caron A (2017). Loss of hepatic DEPTOR alters the metabolic transition to fasting. Mol. Metab..

[46] Hsu PP (2011). The mTOR-regulated phosphoproteome reveals a mechanism of mTORC1-mediated inhibition of growth factor signaling. Science.

[47] Yu Y (2011). Phosphoproteomic analysis identifies Grb10 as an mTORC1 substrate that negatively regulates insulin signaling. Science.

[48] Lee JH (2010). Sestrin as a feedback inhibitor of TOR that prevents age-related pathologies. Science.

[49] Kim JS (2015). Sestrin2 inhibits mTORC1 through modulation of GATOR complexes. Sci. Rep..

[50] Zid BM (2009). 4E-BP extends lifespan upon dietary restriction by enhancing mitochondrial activity in Drosophila. Cell.

[51] Khan NA (2017). mTORC1 regulates mitochondrial integrated stress response and mitochondrial myopathy progression. Cell Metab..

[52] Bartolome A (2017). MTORC1 regulates both general autophagy and mitophagy induction after oxidative phosphorylation uncoupling. Mol. Cell Biol..

[53] Tsang CK (2018). SOD1 phosphorylation by mTORC1 couples nutrient sensing and redox regulation. Mol. Cell.

[54] Kannabiran C, Zeng X, Vales LD (1997). The mammalian transcriptional repressor RBP (CBF1) regulates interleukin-6 gene expression. Mol. Cell Biol..

[55] Hinz M (2002). Nuclear factor kappaB-dependent gene expression profiling of Hodgkin’s disease tumor cells, pathogenetic significance, and link to constitutive signal transducer and activator of transcription 5a activity. J. Exp. Med..

[56] March JT (2018). Targeting TGFbeta signaling to address fibrosis using antisense oligonucleotides. Biomedicines.

[57] Zhao B (2008). TEAD mediates YAP-dependent gene induction and growth control. Genes Dev..

[58] Yimlamai D (2014). Hippo pathway activity influences liver cell fate. Cell.

[59] Shalapour S, Karin M (2015). Immunity, inflammation, and cancer: an eternal fight between good and evil. J. Clin. Invest..

[60] Bose SK, Shrivastava S, Meyer K, Ray RB, Ray R (2012). Hepatitis C virus activates the mTOR/S6K1 signaling pathway in inhibiting IRS-1 function for insulin resistance. J. Virol..

[61] Wang Z, Jin W, Jin H, Wang X (2014). mTOR in viral hepatitis and hepatocellular carcinoma: function and treatment. Biomed. Res. Int..

[62] Kwiatkowski DJ (2002). A mouse model of TSC1 reveals sex-dependent lethality from liver hemangiomas, and up-regulation of p70S6 kinase activity in Tsc1 null cells. Hum. Mol. Genet.

[63] Cho CS (2018). Lipotoxicity induces hepatic protein inclusions through TANK binding kinase 1-mediated p62/sequestosome 1 phosphorylation. Hepatology.

[64] Dobin A (2013). STAR: ultrafast universal RNA-seq aligner. Bioinformatics.

[65] Trapnell C (2012). Differential gene and transcript expression analysis of RNA-seq experiments with TopHat and Cufflinks. Nat. Protoc..

[66] Namkoong S, Ho A, Woo YM, Kwak H, Lee JH (2018). Systematic characterization of stress-induced RNA granulation. Mol. Cell.

