# Supplementary material for: Concurrent activation of growth factor and nutrient arms of mTORC1 induces oxidative liver injury
Source: Cell Discov. 2019 Nov 19;5:60. doi: 10.1038/s41421-019-0131-9 (PMC6868011; doi:10.1038/s41421-019-0131-9)
Supplement: Supplementary file 3 — Supplementary Information [file 41421_2019_131_MOESM3_ESM.pdf]

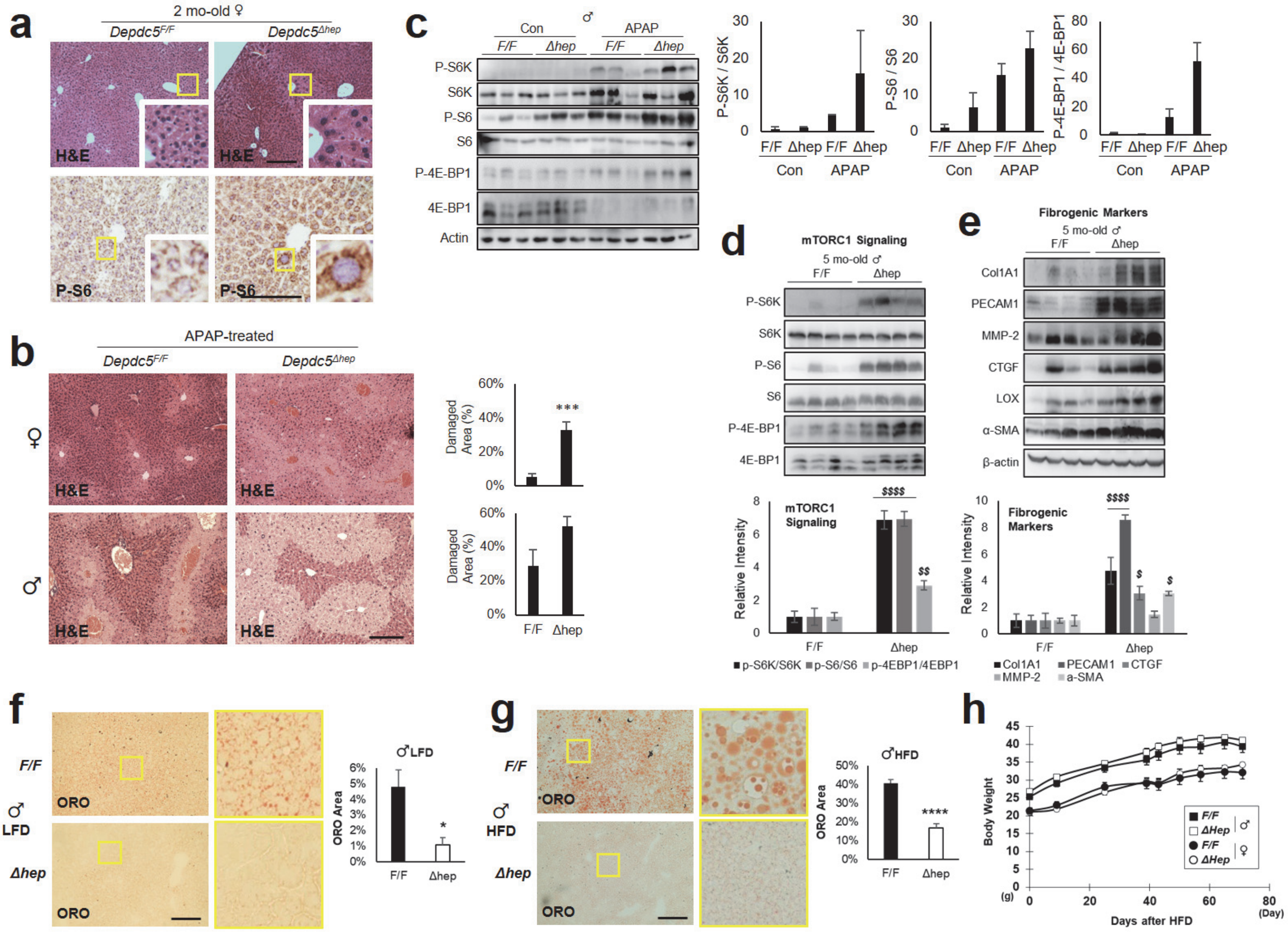


**Fig. S1: Liver phenotypes of *Depdc5*$^{\Delta hep}$ mice. a,** Liver sections were subjected to H&E staining and anti-phospho-S6 immunostaining for 2-month-old females of the Fig. 1 cohort. Boxed areas are magnified in the insets. **b, c,** After 12 hours of fasting, 2-month-old *Depdc5*$^{F/F}$ and *Depdc5*$^{\Delta hep}$ littermate mice of indicated gender were injected with 400 mg/Kg of APAP. After 8 hours, livers were collected and analyzed by H&E staining (**b**) and immunoblotting (**c**). Damaged areas (n≥8) and band intensities (n=3) were quantified. **d, e,** Liver lysates from 5-month-old *Depdc5*$^{F/F}$ and *Depdc5*$^{\Delta hep}$ littermate male mice were subjected to immunoblotting (upper panels) and quantification (lower panels; n=4) to analyze mTORC1 signaling (**d**) and fibrogenic markers (**e**). **f, g,** Two-month-old *Depdc5*$^{F/F}$ and *Depdc5*$^{\Delta hep}$ littermate male mice were kept on normal chow (LFD) (**f**) or high fat diet (HFD) (**g**) for three additional months. Livers from the 5-month-old mice were subjected to Oil Red O (ORO) staining. ORO-positive areas were quantified (right panels, n=3). Boxed areas are magnified in right panels. **h,** Body weight was monitored during HFD feeding (male, n=8; female, n≥4). Data are presented as mean ± SEM. **P* < 0.05; ****P* < 0.001; *****P* < 0.0001(Student's t-test). When multiple parameters were assessed, the Holm-Šídák method was used to compare groups (*$*, *P*<0.05; *$$*, *P*<0.01; *$$$$*, *P*<0.0001). Scale bars, 200 μm.

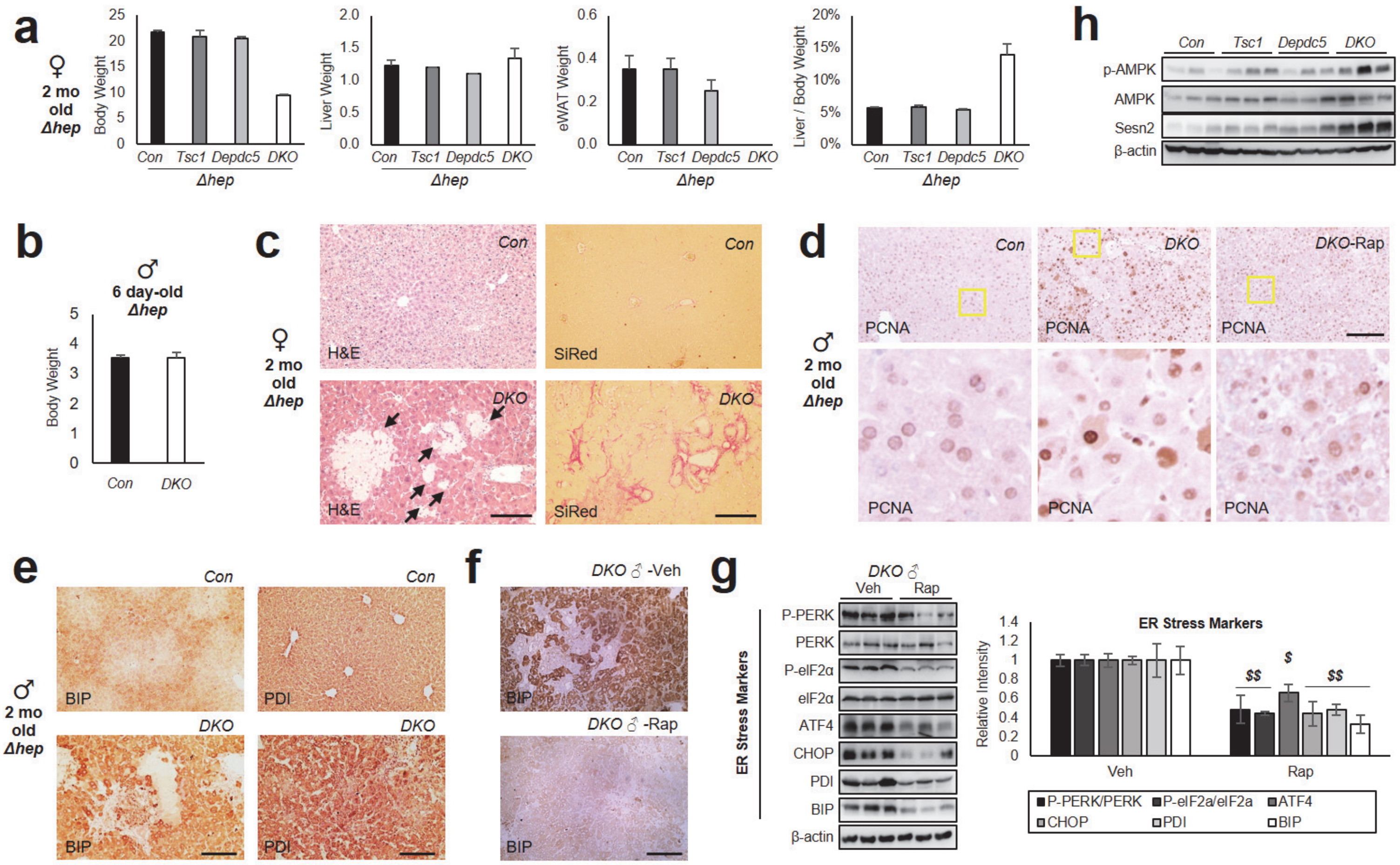


**Fig. S2: DKO mice exhibit mTORC1-dependent liver pathologies. a,** Body, liver, eWAT weights, and liver/body weight ratios were analyzed for females of the Fig. 2 cohort (n≥3 for Con and DKO; n=2 for single knockouts). **b,** Body weights were analyzed for male pups at 6 days after birth (n≥3). **c,** H&E and SiRed staining images from livers of the Fig. 2 female cohort. **d, e,** PCNA (**d**), BIP and PDI (**e**) immunostaining images from livers of the Fig. 2 male cohort. Boxed areas are magnified in bottom panels (**d**). **f,** BIP immunostaining images from livers of the Fig. 4 cohort. **g,** Immunoblotting of ER stress markers (left panel) and quantification (right panel, n=3) for livers of the Fig. 4 cohort. β-actin image is the same as the one presented in Fig. 4f. **h,** Immunoblotting of indicated proteins for livers of the Fig. 3 cohort. Data are presented as mean ± SEM. The Holm-Šídák method was used for multiple comparison tests (*$*, $P<0.05$; *$$*, $P<0.01$). Scale bars, 200 µm.

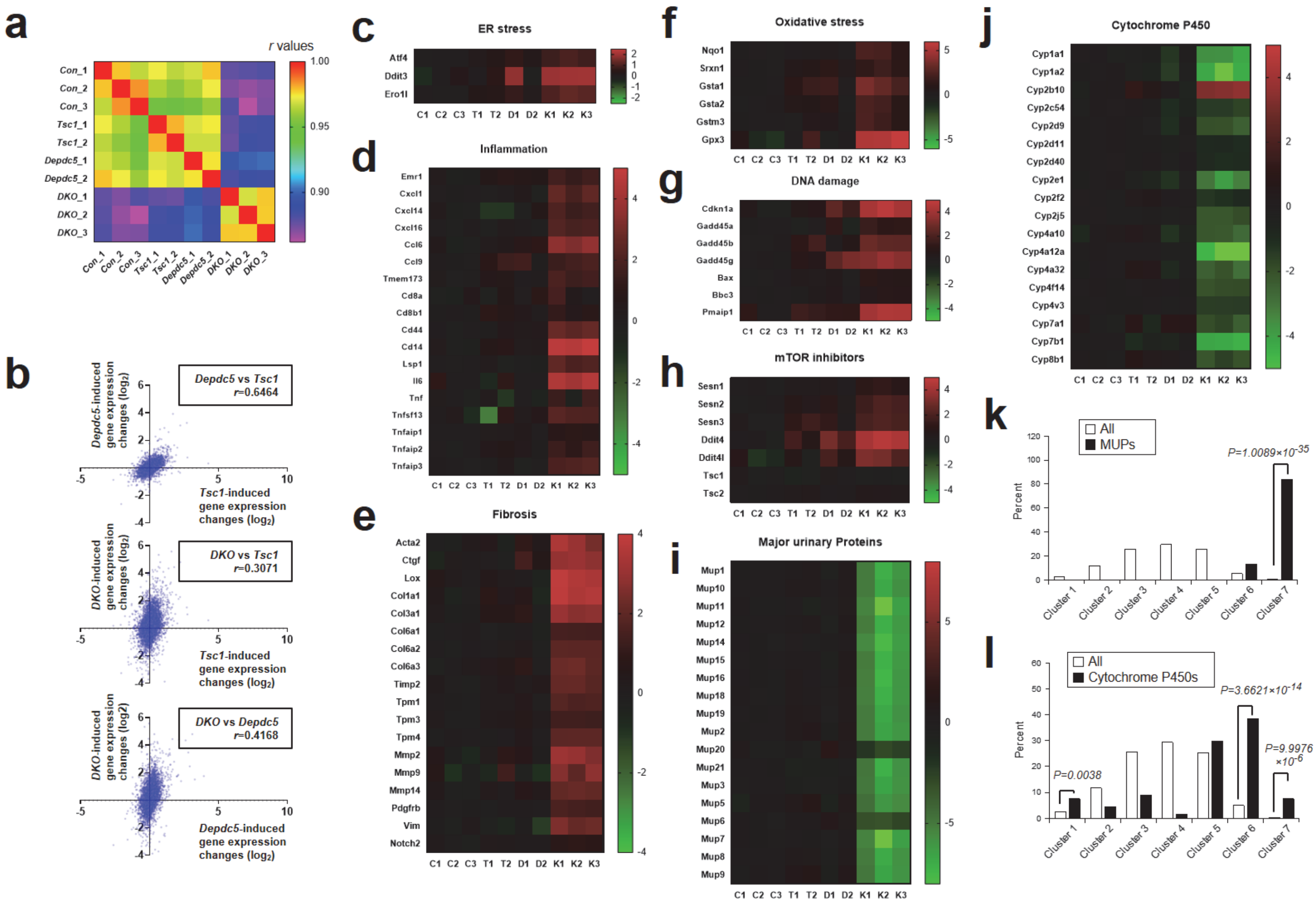


**Fig. S3: The DKO transcriptome profile is distinct from controls and single knockout mutants and is characterized by stress response. a,** Heat map diagram depicting the correlation between individual RNA-seq profiles. **b,** Comparison of mRNA expression fold change over control liver samples between indicated liver samples. Individual dots represent single mRNA species. **c-j,** Heat map diagrams representing mRNA expression fold change from averaged control values (expressed as $log_2$ values). Each column represents a liver sample from a different *Con* (C1-3), *Tsc1*$^{\Delta hep}$(T1-2), *Depdc5*$^{\Delta hep}$(D1-2), or *DKO* (K1-3) mouse. **k,** Gene cluster enrichment analysis of major urinary proteins (MUPs). **l,** Gene cluster enrichment analysis of Cytochrome P450s. Correlations between RNA-seq datasets were assessed by computing a nonparametric Spearman correlation (*r*); *P*<0.0001 for all correlation observations. Statistical significance of gene enrichments in a specific cluster was examined by Fisher's exact test.

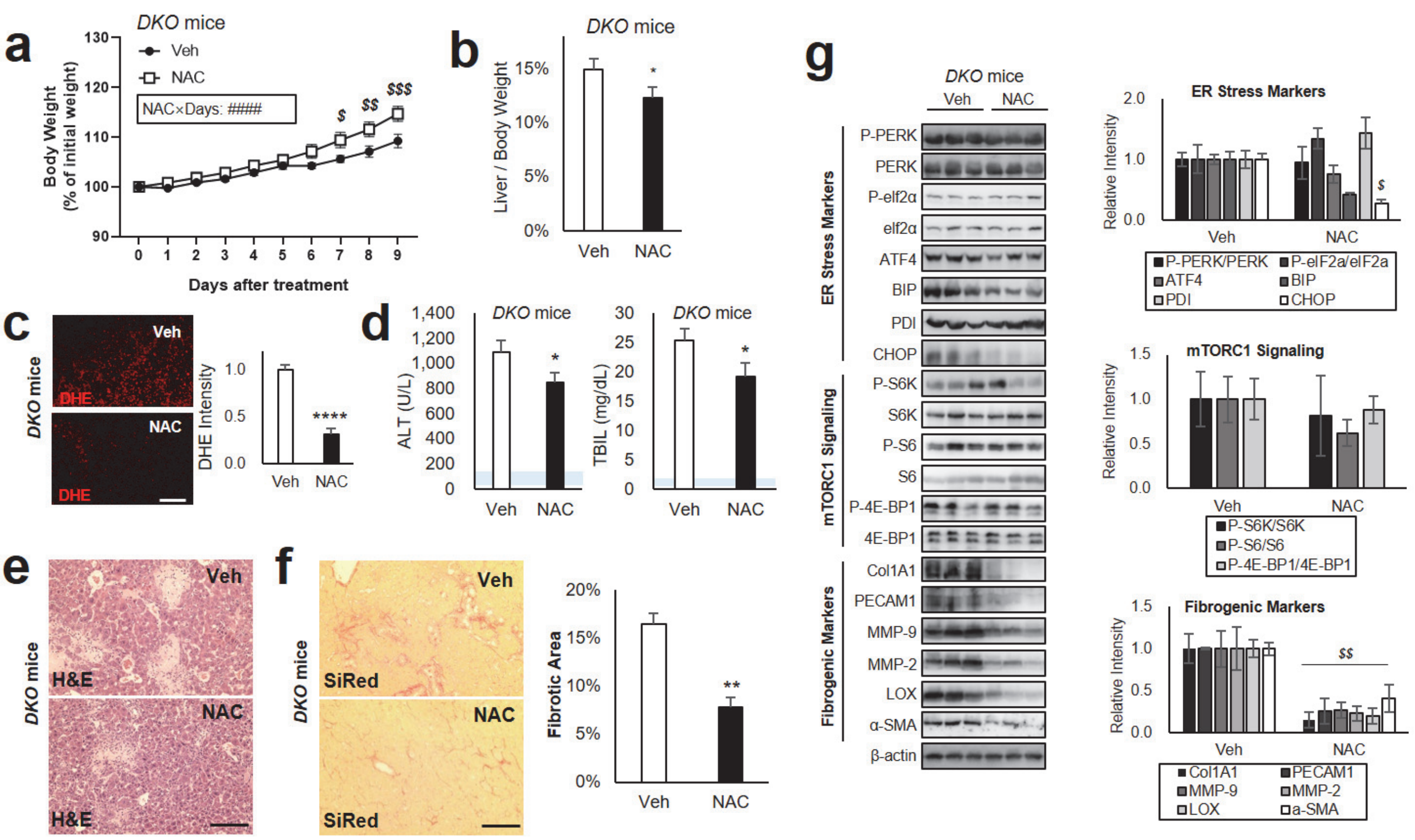


**Fig. S4: N-acetylcysteine (NAC) relieves DKO liver pathologies.** Littermate cohorts of six-week-old DKO mice were injected daily with vehicle (Veh) or NAC (250 mg/kg) for 10 days (n≥10). For drug treatment experiments, mice were gender-matched with both males and females. **a,** Body weight was monitored throughout the course of the experiment. **b,** Liver/body weight ratio was measured at the experimental endpoint. **c,** Dihydroethidium (DHE) staining of liver sections and quantification. **d,** Serum markers for liver damage were analyzed. Blue shaded regions indicate clinically normal ranges. **e,** Liver sections were analyzed by H&E staining. **f,** Liver sections were analyzed by SiRed staining. Fibrotic areas were quantified. **g,** Liver lysates were subjected to immunoblotting (left panels) and quantification (right panels) to examine ER stress markers (top), mTORC1 signaling (middle), and fibrogenic markers (bottom). Data are presented as mean ± SEM. **P* < 0.05; ***P* < 0.01; *****P* < 0.0001(Student's t-test). Interaction between NAC and treatment days (NAC×Days) were assessed through RM two-way ANOVA (####, P<0.0001), and differences in individual data points were assessed through Sidak's multiple comparison test (*$*, *P*<0.05; *$$*, *P*<0.01; *$$$*, *P*<0.001). For western blot quantification, the Holm-Šídák method was used to compare groups (*$*, *P*<0.05; *$$*, *P*<0.01). Scale bars, 200 μm.

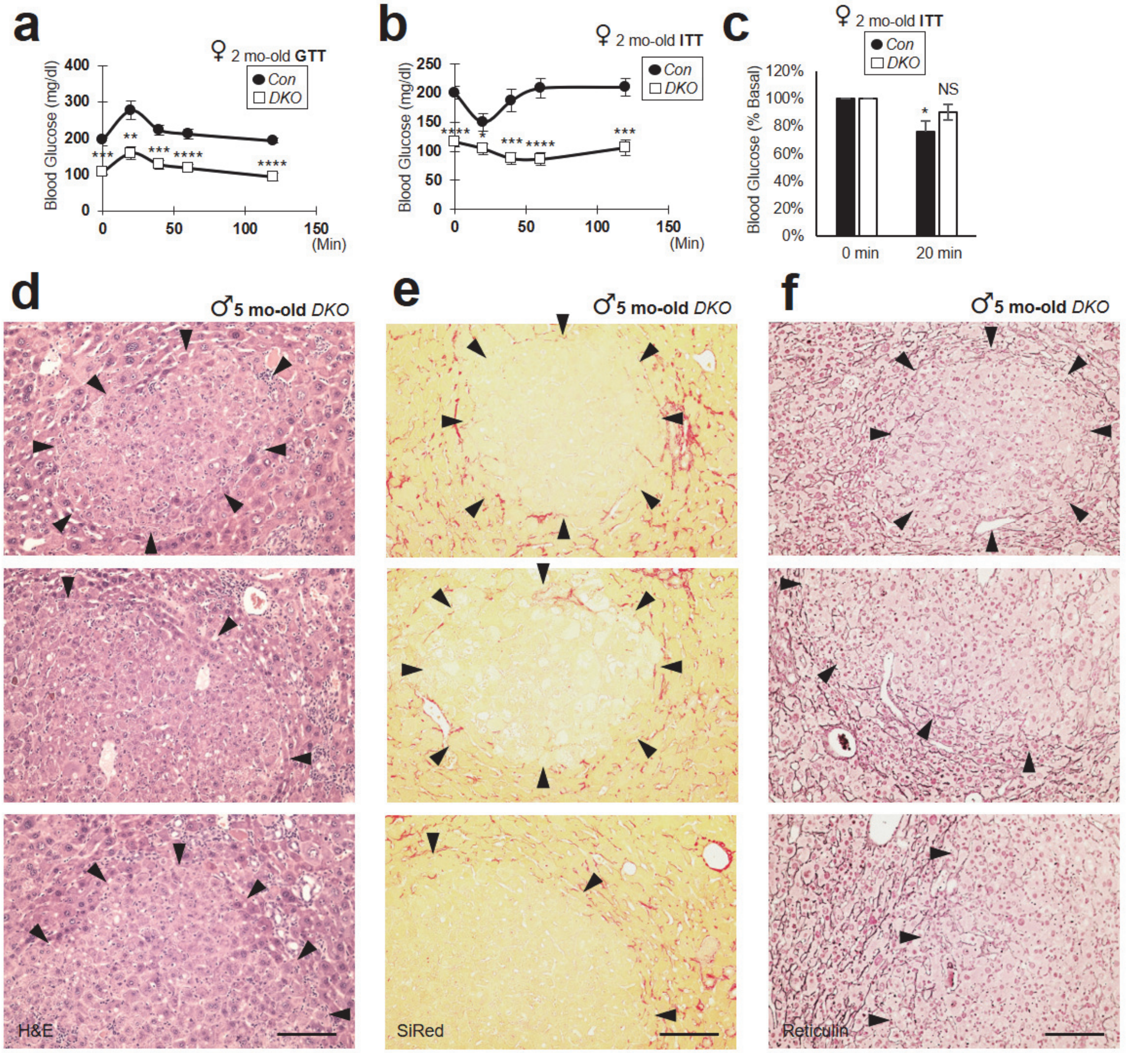


**Fig. S5: *DKO* mice have glucose metabolism defects and HCC development. a-c,** After 4-6 hours of fasting, two-month-old *Con* and *DKO* female littermates (n≥7) were subjected to (**a**) glucose and (**b**) insulin tolerance tests (GTT and ITT, respectively). Data are normalized according to baseline glucose levels (**c**). **d-f,** Additional histology images for Fig. 8e upper right (**d**), 8e middle left (**e**) and 8e middle right (**f**) are presented. Arrowheads indicate approximate boundaries of tumor nodules. Data are presented as mean ± SEM. *$P$<0.05, **$P$<0.01, ***$P$<0.001, ****$P$<0.0001, NS ($P$=0.1050) from a Student's t-test. Scale bars, 200 μm.
